# The Effects of Maternal Intake of EPA and DHA Enriched Diet During Pregnancy and Lactation on Offspring’s Muscle Development and Energy Homeostasis

**DOI:** 10.3389/fphys.2022.881624

**Published:** 2022-06-06

**Authors:** Saeed Ghnaimawi, Shilei Zhang, Jamie I. Baum, Yan Huang

**Affiliations:** ^1^ Medical Laboratory Techniques Department, Kut University College, Alkut, Iraq; ^2^ College of Animal Science and Technology, Shihezi University, Shihezi, China; ^3^ Department of Food Science, Division of Agriculture, University of Arkansas, Fayetteville, AR, United States; ^4^ Department of Animal Science, Division of Agriculture, University of Arkansas, Fayetteville, AR, United States

**Keywords:** maternal, EPA and DHA, muscle, energy metabolism, gestation and lactation

## Abstract

EPA and DHA are n-3 long-chain polyunsaturated fatty acids with a diversity of health benefits on offspring. The objective of this study was to test the *in vivo* effect of maternal ingestion of EPA and DHA on fetal and offspring muscle development and energy balance. Two groups of female C57BL/6 mice were fed EPA and DHA enriched diet (FA) and diet devoid of EPA and DHA (CON) respectively throughout the entire period of gestation and lactation. Embryos at E13 and offspring at age of D1 and D21 were selected for sample collection and processing. No change in birth number and body weight were observed between groups at D1 and D21. Transient increase in the expression levels of myogenesis regulating genes was detected at D1 (*p* < 0.05) in FA group. Most of the expression of muscle protein synthesis regulating genes were comparable (*p* > 0.05) between FA and CON groups at D1 and D21. The significant increase in MHC4, and IGF-1 was not linked to increased muscle mass. A persistent increase in ISR expression (*p* < 0.05) but not in GLUT-4 (*p* > 0.05) was detected in offspring. Up-regulation of adipogenesis regulating genes was accompanied by increasing intramuscular fat accumulation in the offspring of FA group. Considerable increase in transcripts of genes regulating lipid catabolism and thermogenesis in liver (*p* < 0.05) was noticed in FA group at D21; whereas, only the levels of carnitine palmitoyl transferase 1A (Cpt1α) and Enoyl-CoA Hydratase And 3-Hydroxyacyl CoA Dehydrogenase (Ehhadh) increased at D1. Similarly, genes regulating lipolysis were highly expressed at D21 in FA group. EPA and DHA treatment promoted BAT development and activity by increasing the expression of BAT signature genes (*p* < 0.05). Also, maternal intake of EPA and DHA enriched diet enhanced browning of sWAT. Taken together, maternal ingestion of EPA/DHA may be suggested as a therapeutic option to improve body composition and counteract childhood obesity- related metabolic disorders and confer lifelong positive metabolic impact on offspring.

## Introduction

A positive correlation has been suggested between offspring’s health and growth and dietary habit of mothers (Elahi et al., 2009; Mennitti et al., 2015). The quantity and quality of diet consumed by the mothers at pregnancy and lactation are of a great importance for offspring’s growth and development. Obesity is a threat leading factor for both mothers and newborns. Offspring born from obese women are prone to develop many pathophysiological disorders such as asthma, metabolic syndrome, obesity, and impaired neuronal development (Catalano and Ehrenberg, 2006; Segovia et al., 2014). Given the continuous increase in the number of obese women at reproductive age (Yang and Colditz, 2015), an effective maternal nutrition strategy should be launched to protect offspring from multitude of metabolic disorders induced by maternal obesity**.** Anticipating that dietary intervention is more likely a safe approach in regulating energy balance and adiposity compared to using some drugs and its related side effects (Santulli and Iaccarino, 2016), food-derived components known to be involved in enhancing energy expenditure such as Polyunsaturated fatty acids, particularly EPA and DHA can be assumed as excellent options to combat current widespread childhood obesity. Polyunsaturated fatty acids mainly long-chain derivatives (LC-PUFA) are an important partition of diet that are essential in synthesizing the lipid bilayer of cell membrane. Many studies have emphasized the inability of fetus and infant to catalyze critical chemical reactions requisite for synthesizing such fatty acids (Gale et al., 2008; Gil-Sánchez et al., 2011; Marchix et al., 2020). Thus, LC-PUFAs or their precursors should be supplied in sufficient amount in maternal diet to compensate insufficiency. Eicosapetaenoic acid (EPA, 20:5n-3) and docosahexaenoic acid (DHA, 22:6 n-3) are kind of n-3 LC-PUFAs, highly suggested during pregnancy because of their great importance in promoting placental function, growth progression, and neuronal development (Greenberg et al., 2008; Chavan-Gautam et al., 2018). Moreover, the effectiveness of EPA and DHA in stimulating the thermogenic capacity of brown adipocytes have been indicated by several studies (Zhao and Chen, 2014; Bargut et al., 2016a; Kim et al., 2016; Okla et al., 2017; Pahlavani et al., 2017). In accordance with these results, it is conceivable that maternal diet enriched with EPA and DHA could promote prenatal resistance to epidemic childhood obesity associated health problems. However, despite the well-established roles of EPA and DHA in aforementioned metabolic benefits, many studies using *in vitro* models have asserted that EPA and DHA supplementation stimulates fetal myoblast trans-differentiation into white adipocytes-like cells and increased intramuscular lipid infiltration that may lead to developing insulin resistance and muscle atrophy while *in vivo* evidence is still missing (Peng et al., 2012; Hsueh et al., 2018; Ghnaimawi et al., 2019; Zhang et al., 2019; Lacham-Kaplan et al., 2020). Furthermore, accumulative evidences, both *in vivo* and *in vitro*, have been provided to affirm the beneficial roles of EPA and DHA in promoting the thermogenic capacity of brown adipocytes and enhancing the conversion of white to brown adipocyte in adults (Bargut et al., 2016b; Quesada-López et al., 2016; Pahlavani et al., 2017; Ghandour et al., 2018; Worsch et al., 2018; Bargut et al., 2019), whereas their effects on energy regulation in offspring still need to be investigated. Thus, the objective of this study is to elucidate the effect of maternal EPA and DHA supplementation on fetal and offspring’s muscle development and potential metabolic alterations. The study aims at 1) investigating the effect of maternal EPA and DHA enriched diet on muscle development and metabolism 2) investigating the effect of maternal EPA and DHA on adipogenesis and intramuscular lipid accumulation 3) investigating the effect of maternal EPA and DHA on energy balance and fat distribution in weaned mice through studying lipid metabolism in liver, brown adipose tissue (BAT) activity and development, potential browning of sub-cutaneous fat. Considering their high affinity to bind and activation of PPARs family members, we hypothesize that EPA and DHA can promote energy dissipation *via* increasing BAT metabolic activity, browning of subcutaneous fat, and enhancing lipid catabolism in the liver. However, it may increase intramuscular fat accretion through direct effect on genes regulating basal adipogenesis.

## Materials and Methods

### Animals

Animals’ handling and care in this study were approved by Institutional Animal Care & Use Committee (IACUC) in University of Arkansas (Protocol # is 19057R). Forty female, 4–6 weeks old, and forty male C57BL/6J mice obtained from Jackson Laboratory were housed for 2 weeks under constant conditions of temperature and humidity for acclimatization. The mice were exposed to unchanged cycle of 12 h of light and darkness and had an access to food and water ad libitum. Mice were checked daily for estrus cycle, and females identified with typical signs of estrus were housed individually with males for breeding. Once the vaginal plug was observed, females were separated and assigned for one of two types of diets. Animals were fed 3.05% fish oil diet enriched with EPA and DHA (FA) (TD.190782) or control diet (CON), replacing the fish oil with soybean oil (TD.160647, Envigo Madison, Wisconsin United States, www.envigo.com). The mice from different groups were maintained on their respective diet throughout the periods of gestation and lactation. Diets composition are explained in (Table 1). Twenty females were euthanized at day 13 of gestation and two fetuses from each mother were randomly selected for gene expression. Due to the small size of embryos at day 13 of gestation, the whole body of each fetus was collected after removing the head. The other twenty mice were allowed to give birth. On day 1 postpartum, pups were euthanized by CO_2_ inhalation after being weighted. The average number of pups per litter was seven, and only three pups from each litter was selected randomly to be assessed for different parameters. At this time point (D1), livers and all muscles of the thigh were sampled after removing the skin because of the complexity of isolating fetal hindlimb muscles and the insufficiency of individual muscle for RNA and protein extraction. The number of birth and body weights of pups were recorded where the parameters of all the pups per litter were considered in this experiment, and the results of each litter were used for statistical analysis. On the 21st day postpartum, the weaned mice were euthanized using CO_2_ inhalation and samples from quadriceps muscle (vastus intermedius, vastus lateralis, vastus medialis, and rectus femoris muscle), gastrocnemius muscle, subcutaneous WAT, visceral WAT, and BAT were dissected and frozen in liquid nitrogen for gene expression and western blots analysis. Some of the peri-renal fat, tibialis anterior muscles, Subcutaneous WAT, and visceral WAT were collected and fixed in formalin 4% for histology. Twenty pups, selected randomly, one from each litter, were used for different assessments.

**TABLE 1:** Primer sequences for real-time PCR.

Primers	Forward sequence	Reverse sequence
UCP1	TCT​CTG​CCA​GGA​CAG​TAC​CC	AGA​AGC​CAC​AAA​CCC​TTT​GA
PRDM16	AAG​GAG​GCC​GAC​TTT​GGA​TG	TTT​GAT​GCA​GCT​CTC​CTG​GG
MHC	CGC​CCA​CCT​GGA​GCG​GAT​GA	CTT​GCG​GTC​CTC​CTC​GGT​CTG​GT
DIO2	CAG​TGT​GGT​GCA​CGT​CTC​CAA​TC	TGA​ACC​AAA​GTT​GAC​CAC​CAG
IGF1	TCC​TTA​TGA​ATT​GGC​TTA​TC	GTT​TGT​CAT​CTT​CCA​TTC​TGT​T
PGC1α	TCC​TCT​GAC​CCC​AGA​GTC​AC	CTT​GGT​TGG​CTT​TAT​GAG​GAG​G
mTOR	GCC​CAC​GCC​TGC​CAT​ACT​TG	TCA​GCT​CCG​GGT​CTT​CCT​TGT​T
CIDEA	TGCTCTTCTGTATCGCCCAGT	GCCGTGTTAAGGAATCTGCTG
PPARγ	GAT​GTC​TCA​CAA​TGC​CAT​CAG	TCA​GCA​GAC​TCT​GGG​TTC​AG
Atrogin-1	CGTGCACGGCCAACAACC	CCC​GCC​AAC​GTC​TCC​TCA​AT
18S	GTA​ACC​CGT​TGA​ACC​CCA​TT	CCA​TCC​AAT​CGG​TAG​TAG​CG
COX7a1	CAG​CGT​CAT​GGT​CAG​TCT​GT	AGA​AAA​CCG​TGT​GGC​AGA​GA
COX8b	GAACCATGAAGCCAACGACT	GCGAAGTTCACAGTGGTTCC
MuRF1	GGC​TGC​GAA​TCC​CTA​CTG​G	TGA​TCT​TCT​CGT​CTT​CGT​GTT​CCT
α-actin	CAG​AGC​AAG​CGA​GGT​ATC​C	GTC​CCC​AGA​ATC​CAA​CAC​G
Fasn	GCA​TTC​AGA​ATC​GTG​GCA​TA	TTG​CTG​GCA​CTA​CAG​AAT​GC
CPT1α	TAT​AAC​AGG​TGG​TTT​GAC​A	CAGAGGTGCCCAATGATG
LPL	TCT​CCT​GAT​GAC​GCT​GAT​TTT​G	TCT​CTT​GGC​TCT​GAC​CTT​GTT​G
Scd1	GAGGCCTGTACGGGATCATA	CAGCCGAGCCTTGTAAGTTC
Acadvl	CAC​TCA​GGC​AGT​TCT​GGA​CA	TCC​CAG​GGT​AAC​GCT​AAC​AC
Lcad	GGACTCCGGTTCTGCTTCCA	TGCAATCGGGTACTCCCACA
Mcad	CAACACTCGAAAGCGGCTCA	ACTTGCGGGCAGTTGCTTG
Cpt1a	CTC​AGT​GGG​AGC​GAC​TCT​TCA	GGC​CTC​TGT​GGT​ACA​CGA​CAA
Slc25a20	CCG​AAA​CCC​ATC​AGT​CCG​TTT​AA	ACA​TAG​GTG​GCT​GTC​CAG​ACA​A
ATGL	TTC​CCC​AAA​GAG​ACG​ACG​TG	CGG​TGA​TGG​TGC​TCT​TGA​GT
HSL	CCC​TCG​GCT​GTC​AAC​TTC​TT	GGT​GCT​AAT​CTC​GTC​TCG​GG
MGL	ACT​TCT​CCG​GCA​TGG​TTC​TG	GGG​ACA​TGT​TTG​GCA​GGA​CA
Srebp1c	ATCTCCTAGAGCGAGCGTTG	TATTTAGCAACTGCAGATATCCAAG
Ehhadh	AAA​GCT​AGT​TTG​GAC​CAT​ACG​G	ATG​TAA​GGC​CAG​TGG​GAG​ATT
Adrb3	GCTGACTTGGTAGTGGGACTC	TAGAAGGAGACGGAGGAGGAG
Adrb1	CGTCCGTCGTCTCCTTCTAC	CATGATGATGCCCAGTGTCTTG
Slc22a5	TTG​GAG​ACG​AAG​GAC​GGA​CG	GCT​CAG​AGA​AGT​TGG​CGA​TGG
Zic1	CTG​TTG​TGG​GAG​ACA​CGA​TG	CCT​CTT​CTC​AGG​GCT​CAC​AG
FGF21	CAA​ATC​CTG​GGT​GTC​AAA​GC	CAT​GGG​CTT​CAG​ACT​GGT​AC
Ptgs2	CAA​GAC​AGA​TCA​TAA​GCG​AGG​A	GGC​GCA​GTT​TAT​GTT​GTC​TGT
Shox2	TGG​AAC​AAC​TCA​ACG​AGC​TGG​AGA	TTC​AAA​CTG​GCT​AGC​GGC​TCC​TAT
P2RX5	TGA​TAG​TTA​ATG​GCA​AGG​CGG	TTG​TCT​CGG​TAA​AAC​TCG​CTC
PAT2	AGCCACCCCTCTCAATCT	TGC​CTT​TGA​CCA​GAT​GAA​CC
TMEM26	ACC​CTG​TCA​TCC​CAC​AGA​G	TGT​TTG​GTG​GAG​TCC​TAA​GGT​C
TBX1	GGC​AGG​CAG​ACG​AAT​GTT​C	TTG​TCA​TCT​ACG​GGC​ACA​AAG
MYF5	CCT​GTC​TGG​TCC​CGA​AAG​AAC	GAC​GTG​ATC​CGA​TCC​ACA​ATG
MYOD1	TCT​GGA​GCC​CTC​CTG​GCA​CC	CGG​GAA​GGG​GGA​GAG​TGG​GG
MyoG	GCAATGCACTGGAGTTCG	ACG​ATG​GAC​GTA​AGG​GAG​TG
MRF4	GTG​GAC​CCC​TAC​AGC​TAC​AAA​CC	TGG​AAG​AAA​GGC​GCT​GAA​GAC

### RNA Extraction and cDNA Synthesis

RNA was isolated from liquid nitrogen frozen samples using the RNeasy Mini Kit (Qiagen Ltd., United Kingdom) and Direct-zol™ RNA Miniprep Plus. Nanodrop 2000 (Thermo Scientific) was used to measure the concentration and purity of extracted RNA where 260/280 (1.8-2.0) and 260/230 ratio (≥2) were considered as indicators of high-quality RNA. cDNA was synthesized using an iScript™ reverse transcription Supermix kit (Bio-Rad, Richmond, CA, United States) and the reaction condition was set in accordance with the manufacturer instructions. Gene expression was conducted using CFX Connect Real-Time PCR Detection System (Bio-Rad, Richmond, CA, United States). Relative gene expressions were determined using the comparative Ct method and the data of each target gene was normalized to 18 s values in the same sample. The set of primers used in this experiment are explained in (Table 2).

**TABLE 2:** Diet composition.

Product #	Control diet (con) TD.160647		EPA/DHA diet (FA) TD.190782	
Macronutrients	% by weight	% Kcal from	gm	kcal
Protein	18.8	21.4	18.8	21.4
Carbohydrate	51.6	58.7	51.6	58.7
Fat	7.8	19.9	7.8	19.9
**Energy density**	**3.5**		**3.5**	
Fatty acid composition %*				
Myristic acid C14:0	0		∼3.6	
Palmitic acid (PA) C16:0	∼11.2		∼13.1	
Palmitoleic acid C16:1 (n-7)	0.0		∼4.8	
Stearic acid C18:0	∼3.9		∼3.5	
Oleic acid C18:1 (n-9)	∼23.2		∼18.2	
Linoleic acid C18:2 (n-6)	∼53.5		∼33.2	
Linolenic acid C18:3 (n-3)	∼7.5		∼4.9	
Eicosapentaenoic acid C20:5 (n-3)	0		∼5.7%	
Docosahexaenoic acid C22:6 (n-3)	0		∼4.5%	
SFA	∼15.4%		∼22%	
MUFA	∼23.4%		∼24.7%	
PUFA	∼61.2%		∼53.3%	
N-6 level	∼53.5%		∼36%	
N-3 level	∼7.6%		17.2%	
(n-6): (n-3) ratio	7.1		2.1	

### Western Blot Assay

Samples were prepared for protein extraction by homogenizing ∼100 mg of frozen tissues with MagNA lyser green beads in lysis buffer composed of a mixture of T-PER (Sigma-Aldrich, St. Louis, MO, United States) and protease and phosphatase cocktail inhibiter at ratio of 100:1. The homogenized tissue lysate was transferred into 1.5 tubes and centrifuged at 13,000 g for 20 min. Thereafter, the supernatant containing total proteins was gently aspirated into new centrifuged tubes. The Pierce™ BCA Protein Assay Kit (Sigma-Aldrich, St. Louis, MO, United States) was used to determine the concentration of total protein by following the instructions from manufacturer. Gel electrophoresis was performed by loading 40 μg protein from all samples (control and treatment) into the wells of Mini-PROTEAN precast gels (Bio-Rad). Then, the protein was transferred on the membrane using Trans-Blot ^®^ Turbo™ Mini PVDF Transfer Packs (Bio-Rad). The membrane was blocked for 1 h in 5% blocking solution (dry milk or/and BSA) at room temperature with gentle shaking. After being washed three times with 1X TBST buffer, membrane was incubated overnight with specific primary Abs including:

[glyceraldehyde-3-phosphate dehydrogenase (GAPDH) (1:1000, Cell Signaling), myogenic differentiation 1 (MyoD1) (1:1000, Abcam), Myogenin (MyoG) (1:2000, Abcam), PPARG Coactivator 1 Alpha (PGC1α) (1:1000, Cell Signaling), phosphorylated Mechanistic Target Of Rapamycin Kinase (P-mTOR) (1:1000, Cell Signaling), P-p70s6 kinase (1:1000, Cell Signaling), CPT1α (1:1000, Cusabio), phosphorylated Hormone-sensitive lipase (P-HSL) (1:1000, Abcam), Uncoupling protein 1 (UCP1) (1:1000, Abcam), and PRDM16 (1:1000, Abcam) ]. Then, the membrane was incubated with HPR conjugated secondary antibody diluted to (1:5000) for 1 h at room temperature. ECL immunoblotting clarity system (Bio-Rad) was used to visualize the bands, detected under ChemiDoc TM Touch imaging system (Bio-Rad, California, United States). Band density was analyzed using Image Lab software (Bio-Rad, California, United States) and normalized according to the glyceraldehyde-3-phosphate dehydrogenase (GAPDH) content (Iwase et al., 2020).

### Histology

The whole muscles of the thigh of new born pups (D1) and the tibialis anterior from weaned mice (D21) were collected and stored in 4% paraformaldehyde for subsequent hematoxylin - eosin (H&E) and oil red O staining. Samples stained with hematoxylin and eosin (H&E) were used to determine the number of nuclei per myotube, myotubes’ length, and myotubes’ number and thickness. Further, sections stained with oil red O were used to evaluate intramuscular lipid accumulation. Peri-renal fat pads collected from weaned mice were maintained in 4% paraformaldehyde and stained with hematoxylin and eosin (H&E) to be used in determining adipocyte sizes and numbers. Subcutaneous samples were maintained in 4% paraformaldehyde and stained with hematoxylin and eosin (H&E) to examine the potential browning of white adipocytes. Eight samples were used in each group, and images of eight cross sections from each sample were analyzed. The image analyzing software, Image J (Fiji-win32), was used to analyze all the data. Intramuscular lipid accumulation was identified as a bright red area located in the sarcoplasm of the fibers and/or in endomysium (between fibers). Zeiss Imager M2 microscope and digital camera (Axiocam 105 color)” was used for taking images.

### Hematoxylin and Eosin Staining

Samples were kept in 10% buffered formalin solution until time of processing. Tissue samples were sliced into 2–3 pieces and placed in VWR brand Monosette IV cassettes (Missouri, United States). Tissues were fixed overnight using a Lipshaw Model 2500 automated processor (Thermo Scientific, Waltham, MA, United States). The tissue samples were rinsed in 70%, 80%, 85%, 95% (×2) and 100% (×2) alcohol followed by two rinses in toluene and two paraffin baths. The following morning, tissue samples were removed from the Lipshaw 2500 and embedded in liquid paraffin. After setting up, the paraffin blocks were removed from the forms and placed in a 5F° freezer overnight. Samples were removed from the freezer, one at a time, and cut into ∼2 nm slices using a Lipshaw manual rotary microtome and placed on VWR Superfrost Plus microscope slides. Tissues were placed in a Lipshaw slide dryer overnight. Paraffin was removed from the tissue samples with two toluene rinses followed by 100% (×2), 95% (×2) and tap water rinse before being stained with VWR Harris Hematoxylin solution for 15 min. After rinsing, samples were dipped in an acid alcohol solution 4 times before being rehydrated in dd H_2_O for 15 min. After rehydrating, they were submerged in Harleco brand staining blueing solution for 30 s, followed by 2 min in Eosin Y solution. Samples were then rinsed in alcohol 4 times and toluene 4 times. After the final toluene rinse, cover slips were fixed using Thermo Scientific Xylene Substitute Mountant and left to dry overnight (Sato et al., 2020).

### Oil Red O Staining

Oil red O staining was conducted according to (Luna, 1992). Briefly, 0.5 gm oil red O was gradually dissolved in 100 ml absolute propylene glycol (100%) to prepare working solution (0.5%). Large undissolved pieces were allowed to melt progressively by periodic stirring. The mixture was, thereafter, heated on hot plate with gentle stirring, taking in account the gradual increase of temperature till reaching 95°C as a maximum without exceeding the 100°C. Before getting cold, the solution was filtered directly with rough filter paper. Then, the leached solution was collected in conical 50 ml tube and let stand at room temperature overnight followed by vacuum filtration using Seitz filter with the coarse surface facing up. Muscle samples were preserved in 10% buffered formalin prior to paraffin embedding. Paraffin samples were prepared following the same steps outlined in H&E protocol. Paraffin embedded tissue sections were cut approximately 10 μm thick and mounted on slides. The following day, paraffin was removed from tissue samples with 2 changes xylene (2 min each) followed by 100% ethanol (2 changes/2 min each), 95% reagent alcohol (2 changes/2 min each) and tap water rinse (1 time/1 min). The slides were then placed in Oil Red O working solution (0.5%) for 24 h. Thereafter, they were immersed in 85% propylene glycol for 1 min with gentle agitation. Later, the slides were rinsed in distilled water (2 changes/2 min each), followed by incubation with Mayer’s hematoxylin for 5 min. Afterwards, the slides were rinsed under tap water for 10 min. Finally, Shandon Xylene Subsitute Mountant (Thermo Scientific, Waltham, MA, United States) was used to mount cover glass.

### Statistical Analysis

All data from assays used to compare CON and FA treated groups were assessed for significance by the unpaired Student’s t-test with the assumption of equal variances, and arithmetic means ± SEM are reported. *p <* 0.05 was considered statistically significant.

## Results

### Birth Outcomes and Tissue Weights

**FIGURE 1:**
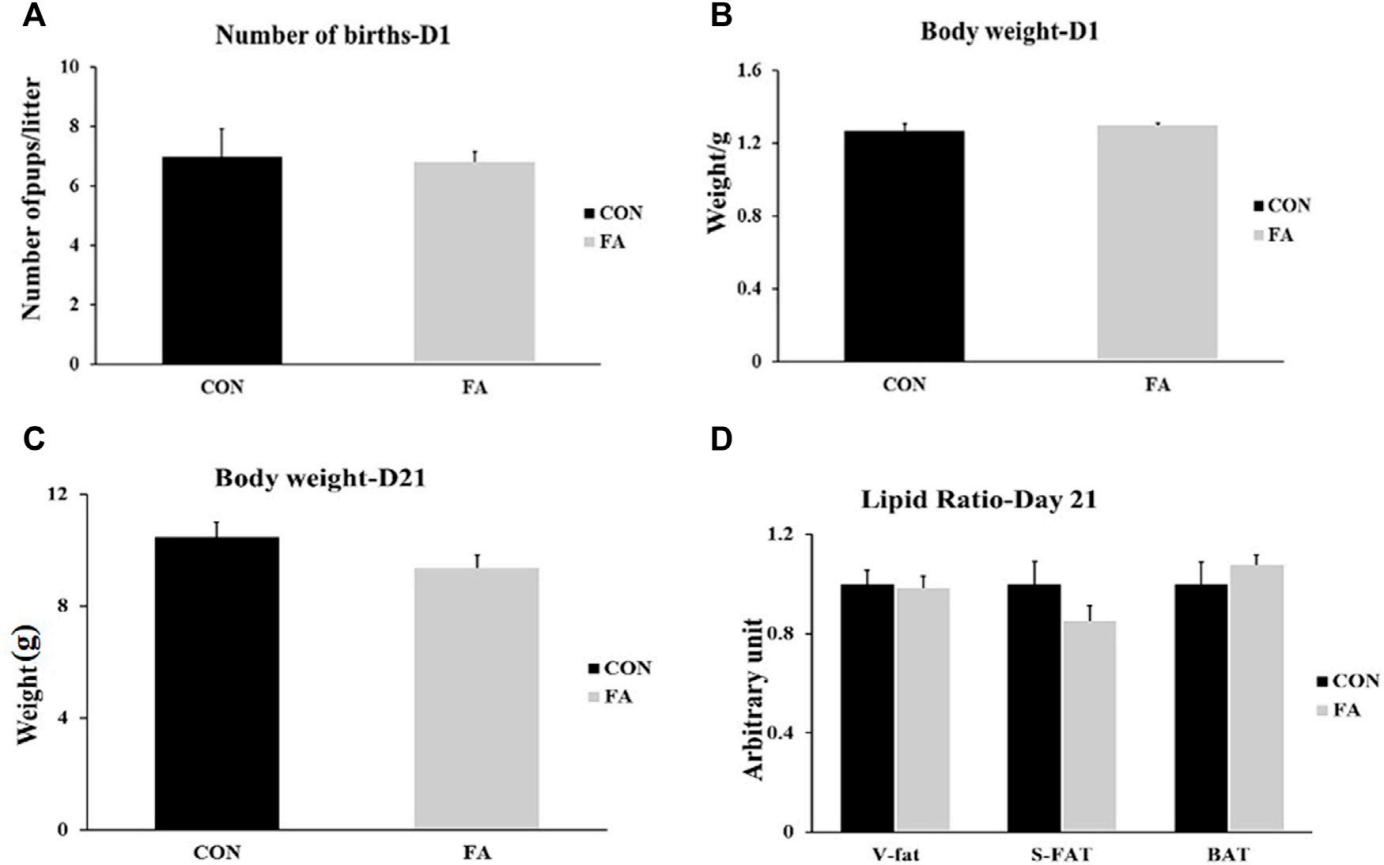
Effect of maternal intake of EPA/DHA enriched diet on number of births and body weight of new born pups at D1 and D21. The mothers were assigned to two groups, one received control diet (CON) and the other was fed EPA/DHA enriched diet (FA). **(A)** Statistical analysis of number of births **(B)** Body weight difference between tested groups at D1. **(C)** Body weight difference between tested groups measured in gram at D21. **(D)** Fat ratio corrected by body weight. Body weight was measured in gram. Data was analyzed by Student’s t-test (*n* = 20) and represent as mean ± SEM. Non-significant difference was observed between groups. Fat ratio was calculated by dividing the weight of a certain fat deposits (BAT, visceral fat, and subcutaneous fat) on total body weight and the results was normalized by body weight.

Our results showed that the number of births and body weight of pups at D1 were not affected by FA treatment (*p* = 0.413476 and 0.252995 respectively). Similarly, although it was not significantly different, FA treated group exhibited a tendency toward decrease body weight (9.36 ± 0.45 g) when compared to control group at weaning (day 21) (10.48 ± 0.53 g) (*p* = 0.062). In line with that, no significant differences were detected in the ratios of different fat depots including subcutaneous fat, visceral fat, and BAT (*p* = 0.08897, 0.078182, 0.218379 respectively) among weaned pups coming from mothers fed control or fatty acids enriched diet (Figure 1).

### Genes Regulating Myogenesis, Muscle Contractile Proteins, Muscle Protein Synthesis, and Adipogenesis at E13

**FIGURE 2:**
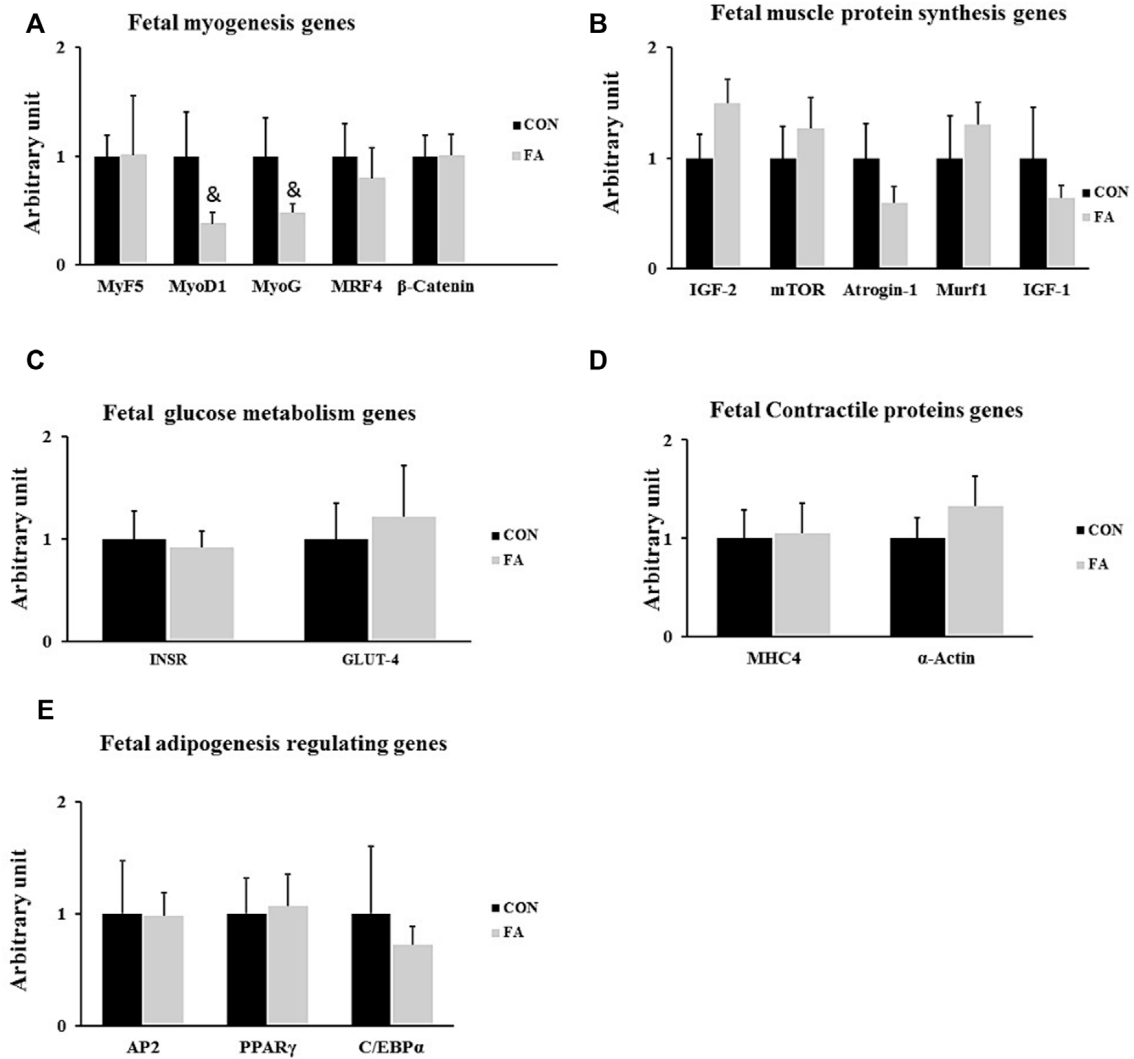
Effect of maternal intake of EPA/DHA on fetal genes regulating myogenesis, muscle protein synthesis, glucose metabolism, muscle contraction, and adipogenesis on day 13 of gestation. The mothers were assigned to two groups, one received control diet (CON) and the other was fed EPA/DHA enriched diet (FA) **(A)** qPCR analysis of genes regulating myogenesis **(B)** qPCR analysis of genes encoding muscle protein synthesis and degradation **(C)** The relative expression of glucose metabolism regulating genes. **(D)** qPCR analysis of genes regulating muscle contractile proteins. **(E)** The relative expression of adipogenesis regulating genes of fetal muscles collected on day 13 of gestation. The Ct values of target genes were normalized to the values of 18 s in each sample. All data represent as mean ± SEM. *p* < 0.05, by Student’s t-test (*n* = 6). The symbol (&) means tendency to significant (0.05 < *p* < 0.1).

The results exhibited non-significant differences between control and FA treated groups in expression levels of genes regulation myogenesis process including MyoD1, MyoG, and Myf5, myogenic regulatory factor (MRF4), and β-catenin (*p* = 0.083881, 0.09182, 0.49113, 0.312692, and 0.494429 respectively). Also, the transcripts of genes encoding the main muscle structural proteins including myosin heavy chain-4 (MHC4) and α-actin were comparable between control and FA treated groups (*p* = 0.448238 and 0.201322 respectively). Moreover, non-significant differences were observed in expression levels of genes regulating muscle protein synthesis and glucose metabolism, including mammalian target of rapamycin (mTOR), muscle ring finger 1 (MURF1), insulin-like growth factor 2 (IGF-2), F-box only protein 32 (Atrogin-1), insulin-like growth factor 1 (IGF-1), Insulin receptor (INSR), and glucose transporter-4 (*p* = 0.26, 0.25, 0.067, 0.13, 0.23, 0.399, and 0.29 respectively). Furthermore, no significant differences were observed in the expression levels of adipogenesis regulating genes including peroxisome proliferator-activated receptor gamma (PPARγ), CCAAT Enhancer Binding Protein Alpha (CEBPα), and Adipocyte fatty acid binding protein (AP2) between control and FA treated groups at this time point (*p* = 0.339223, 0.334623, and 0.48406 respectively) (Figure 2).

### Genes Regulating Myogenesis, Muscle Contractile Proteins and Muscle Protein Synthesis at D1

**FIGURE 3:**
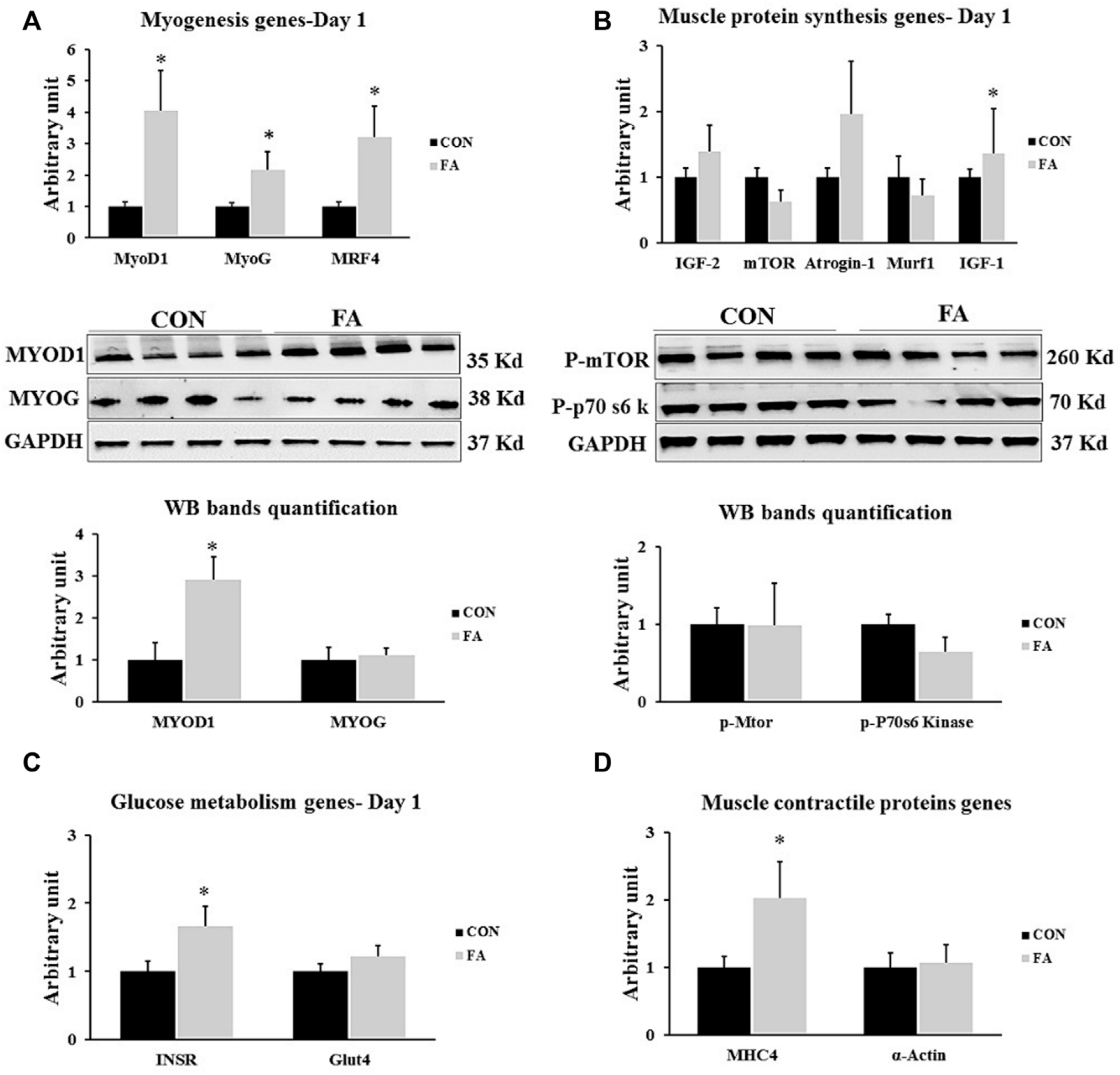
Effect of maternal intake of EPA/DHA on genes regulating myogenesis, muscle protein synthesis, glucose metabolism, muscle contraction, and adipogenesis in day 1 neonates. The mothers were assigned to two groups, one received control diet (CON) and the other was fed EPA/DHA enriched diet (FA) **(A)** Quantitative RT-qPCR (*n* = 8) and representative image and densitometric analysis of western blot (*n* = 4) of genes regulating myogenesis **(B)** Quantitative RT-qPCR (*n* = 8) and representative image and densitometric analysis of western blot (*n* = 4) of genes encoding muscle protein synthesis **(C)** The relative expression of glucose metabolism regulating genes (*n* = 8). **(D)** qPCR analysis of genes regulating muscle contractile proteins (*n* = 8). The raw Ct was normalized to the value of 18 s. All data represent as mean ± SEM. *p* < 0.05 by Student’s t-test.

The results exhibited a considerable increase in the expression level of genes regulation myogenesis process including MyoD1, MyoG, and MRF4 in FA treated group when compared to control (220 ± 81.7% *p* = 0.019004, 115 ± 59.6% *p* = 0.044065, and 221 ± 98.5% *p* = 0.025263 respectively). However, no significant difference was observed in MyoG protein level between differentially treated groups. Also, the transcript of gene encoding MHC4 but not α-actin was significantly higher in FA groups upon comparison to control group (102 ± 54.6% *p* = 0.050957, *p* = 0.427226 respectively). Additionally, our findings revealed a considerable increase in the expression level of IGF-1 in FA group when compared to control group (126 ± 68.5% *p* = 0.018595). However, the results of other genes regulating protein synthesis such as mTOR, MURF1, IGF-2, and Atrogin-1 were comparable between variously treated groups (*p* = 0.276066, 0.261989, 0.193612, and 0.137308 respectively). Also, a significant increase in the expression level of gene encoding insulin receptor (INSR) was detected in FA group in comparison to control group (65 ± 29.3% *p* = 0.037078). However, no significant difference of the expression level of GLUT-4 (P + 0.135904) was observed between the groups. The results may be an indicator of a positive correlation between EPA and DHA supplementation and muscle insulin sensitivity (Figure 3).

### Myotube Formation at D1

**FIGURE 4:**
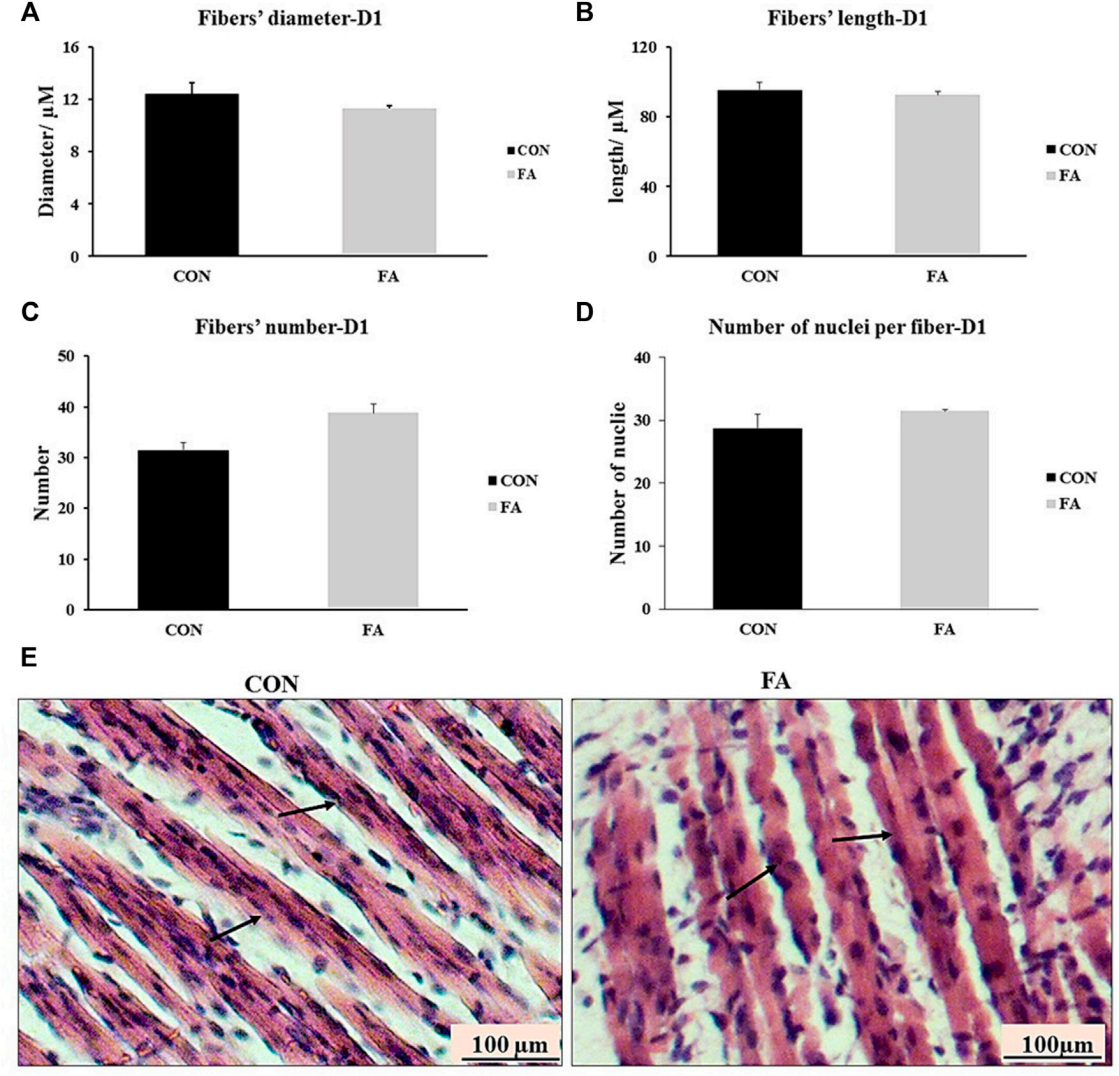
Effect of maternal intake of EPA/DHA on myotubes formation in day 1 neonates. The mothers were fed either control diet (CON) or EPA/DHA enriched diet (FA) during the entire period of pregnancy and lactation. **(A)** Data shows the difference in the diameter of muscle fibers measured in micrometer between CON and FA groups. **(B)** Data shows the difference in the length of muscle fibers measured in micrometer between CON and FA groups. **(C)** The difference in the number of muscle fibers in the cross-sections between CON and FA groups. Images were analyzed in ImageJ-FIJI using the Freehand Selection Tool to encircle individual myofibers. **(D)** The difference in the number of nuclei per muscle fiber between CON and FA groups. The data represent as mean ± SEM. *p* < 0.05 (*n* = 8); eight samples per group were used in this measurement where the value of each samples represents the average of the measurements of eight cross section areas randomly selected from each sample **(E)** a representative microscopic image of muscle tissue stained with H&E from differentially treated groups. The magnification is ×10 and scale bar is 100 μm black arrows refer to muscle fibers. Non-significant difference was observed between groups.

The histological analysis of muscle tissue exhibited non-significant differences in the number of muscle fibers, diameter of fibers, length of fibers, and number of nuclei per fiber between control and FA treated groups in neonates aged 1 day (*p* = 0.399, 0.091, 0.309691, and 0.134535 respectively) (Figure 4). The results were inconsistent with those obtained from gene expression indicating that maternal EPA and DHA intake has no effect on muscle tissue mass and growth in offspring.

### Intramuscular Fat Accumulation at D1

**FIGURE 5:**
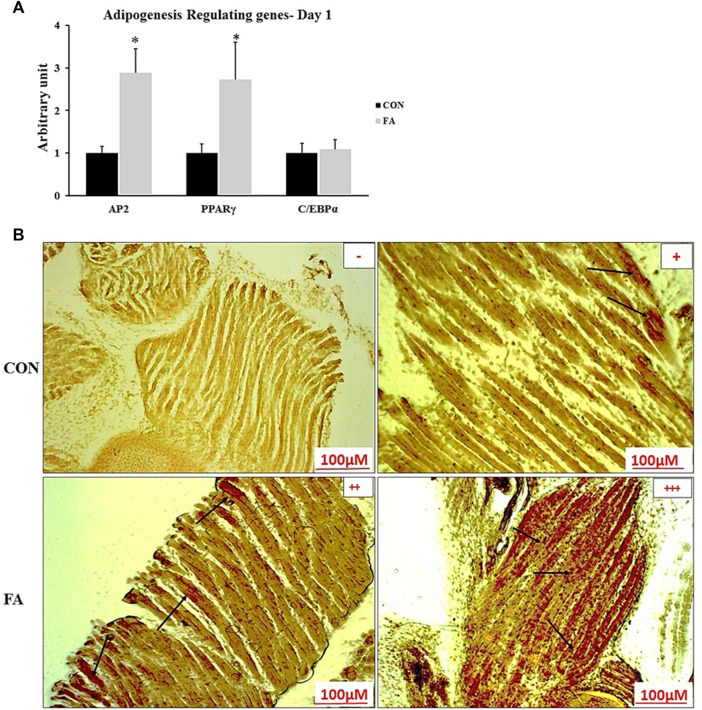
Effect of maternal intake of EPA/DHA on ectopic lipid accumulation in muscles of day 1 neonates. The mothers were fed either control diet (CON) or EPA/DHA enriched diet (FA) during the entire period of pregnancy and lactation. **(A)** The relative expression of adipogenesis regulating genes in neonatal muscles. The raw Ct was normalized to the value of 18 s. All data represent as mean ± SEM. *p* < 0.05 by Student’s t-test (*n* = 8). **(B)** A representative microscopic image of muscle samples stained with Oil red O in day 1 offspring fed control diet or EPA/DHA rich diet. The magnification is ×10 and scale bar is 100 μm. Black arrows refer to accumulated fat. (−) indicates absence the accumulation of fat neither in the sarcoplasm of the fibers nor in endomysium (between fibers). (+) represents mild fat infiltration (++) indicates a moderate load. (+++) indicates an overload of fat.

FA treatment considerably enhanced the expression levels of adipogenesis regulating genes at D1. The expression levels of PPARγ and AP2 were by far much higher in FA treated group when compared to control group (173 ± 86.4% *p* = 0.04064, 187 ± 56.3% *p* = 0.004606 respectively). However, the expression level of CEBPα was not significantly different between the groups (*p* = 0.393167). In line with that, Oil red O-stained sections showed a dramatic increase in ectopic intramuscular accumulation of lipid in FA treated group in comparison to control group in neonates (D1). The results of histology were consistent with PCR results referring to the adipogenic role of EPA and DHA (Figure 5).

### Genes Involved in Lipid Metabolism Regulation in Liver at D1

**FIGURE 6:**
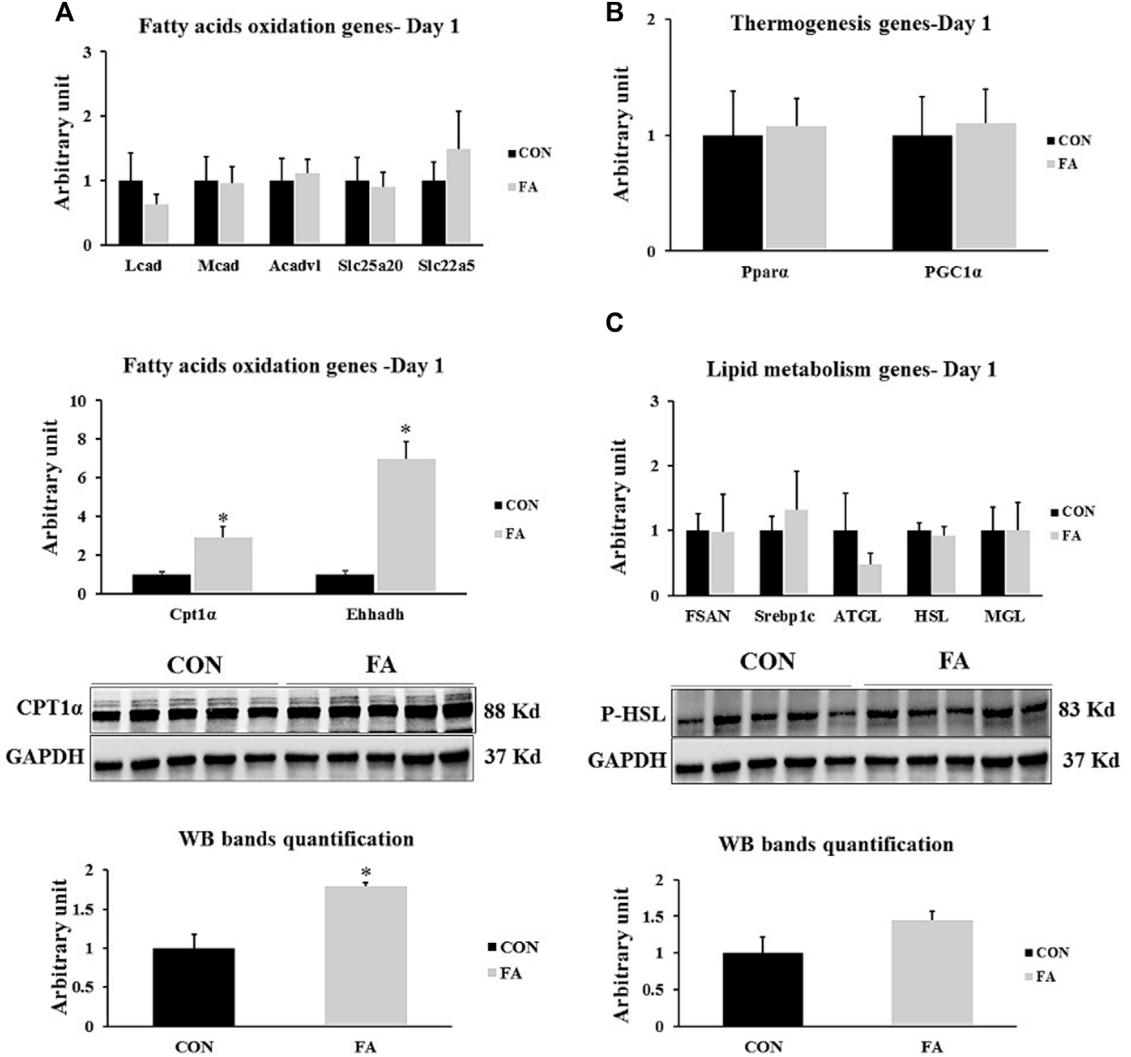
Effect of maternal intake of EPA/DHA on lipid metabolism regulation in liver in day 1 neonates. The mothers were fed either control diet (CON) or EPA/DHA enriched diet (FA) during the entire period of pregnancy and lactation **(A)** Quantitative RT-qPCR (*n* = 8) and representative image and densitometric analysis of western blot (*n* = 5) of genes regulating fatty acid uptake and a β-oxidation (*n* = 8) **(B)** The relative expression of genes involved in regulating the thermogenesis process in liver (*n* = 8). **(C)** Quantitative RT-qPCR (*n* = 8) and representative image and densitometric analysis of western blot (*n* = 4) of genes regulating lipogenesis and lipolysis. The control image of GAPDH from liver samples (D1) was re-used. The raw Ct was normalized to the value of 18 s. All data represent as mean ± SEM. *p* < 0.05 by Student’s t-test.

Obvious partial increase in the expression level of genes regulating fatty acid uptake and β-oxidation CPT1α and Ehhadh was observed in FA treated group in comparison to control group (191 ± 56.9% *p* = 0.004253, 595 ± 89.7% *p* = 0.00003 respectively). However, the expression level of medium-chain acyl-CoA dehydrogenase (Mcad), acyl-CoA dehydrogenase, long chain (Lcad), very long-chain specific acyl-CoA dehydrogenase (Acadvl), mitochondrial carnitine/acylcarnitine carrier protein (Slc25a20)**,** and Solute carrier family 22 member 5 (SLC22A5) showed a great similarity between the differentially treated groups (*p* = 0.464667, 0.219827, 0.396513, 0.413866, and 0.219612 respectively). Moreover, non-significant differences were observed in expression level of thermogenesis regulating genes including peroxisome proliferator-activated receptor gamma coactivator 1-alpha (PGC-1α) and peroxisome proliferator-activated receptor alpha (PPARα) (*p* = 0.409632 and 0.437284 respectively). Our findings also exhibited non-significant differences between tested groups in genes known to be involved in orchestrating lipid synthesis such as fatty acid synthase (Fasn) and sterol regulatory element-binding transcription factor 1 (Srebp1c) (*p* = 0.481307 and 0.315629 respectively). Further, the transcripts of lipolysis regulation genes including adipose triglyceride lipase (ATGL), hormone-sensitive lipase (HSL), and monoacylglycerol lipase (MGL) were comparable between FA and control treated groups at this time point (*p* = 0.200105, 0.34237, and 0.494122 respectively) (Figure 6)**.**

### Genes Regulating Myogenesis, Muscle Contractile Proteins and Muscle Protein Synthesis at D21

**FIGURE 7:**
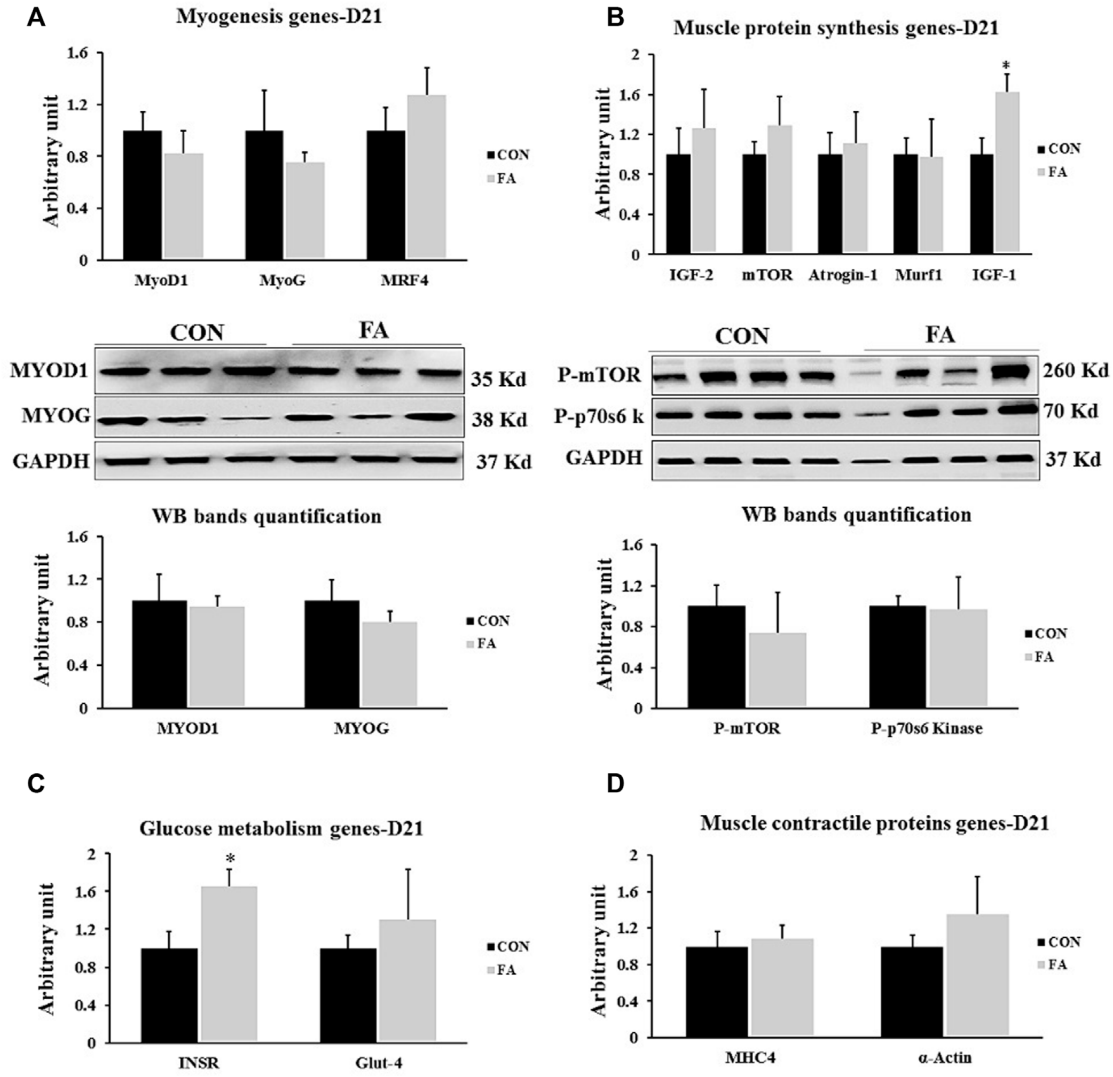
Effect of maternal intake of EPA/DHA on genes regulating myogenesis, muscle protein synthesis, glucose metabolism, and muscle contractile proteins in weaned mice (21 days post-parturition). The mothers were assigned to two groups, one received control diet (CON) and the other was fed EPA/DHA enriched diet (FA) **(A)** Quantitative RT-qPCR and representative image and densitometric analysis of western blot of genes regulating myogenesis **(B)** Quantitative RT-qPCR (*n* = 8) and representative image and densitometric analysis of western blot (*n* = 4) of genes encoding muscle protein synthesis **(C)** The relative expression of muscle glucose metabolism regulating genes. **(D)** qPCR analysis of genes regulating muscle contractile proteins. The raw Ct was normalized to the value of 18 s. All data represent as mean ± SEM. *p* < 0.05 by Student’s t-test.

Non-significant differences between control and EPA and DHA treated groups were observed in the expression levels of genes regulation myogenesis process including MyoD1, MyoG, and MRF4 (*p* = 0.22122668, 0.26232362, and 0.17121615 respectively). Similarly, the transcripts levels of MHC4 and α-actin, genes encoding the main muscle structural proteins were comparable between control and FA groups (*p* = 0.358706 and 0.180359 respectively). Moreover, our findings exhibited a considerable increase in the expression level of IGF-1 in FA treated group upon comparison to control group (62 ± 18%, *p* = 0.01548417). However, the results of other genes regulating protein synthesis such as mTOR, MURF1, IGF-2, and Atrogin-1 were comparable between the groups (*p* = 0.164394, 0.473528, 0.27987, and 0.27987 respectively). Also, a significant increase in the expression levels of gene encoding insulin receptor (INSR) was detected in FA treated group in comparison to control group (64 ± 18.9% *p* = 0.01789307). Further, the expression level of glucose transporter-4 (GLUT-4) exhibited a tendency toward an increase in FA treated groups in (*p* = 0.07259825) (Figure 7).

### Myotube Formation at D21

**FIGURE 8:**
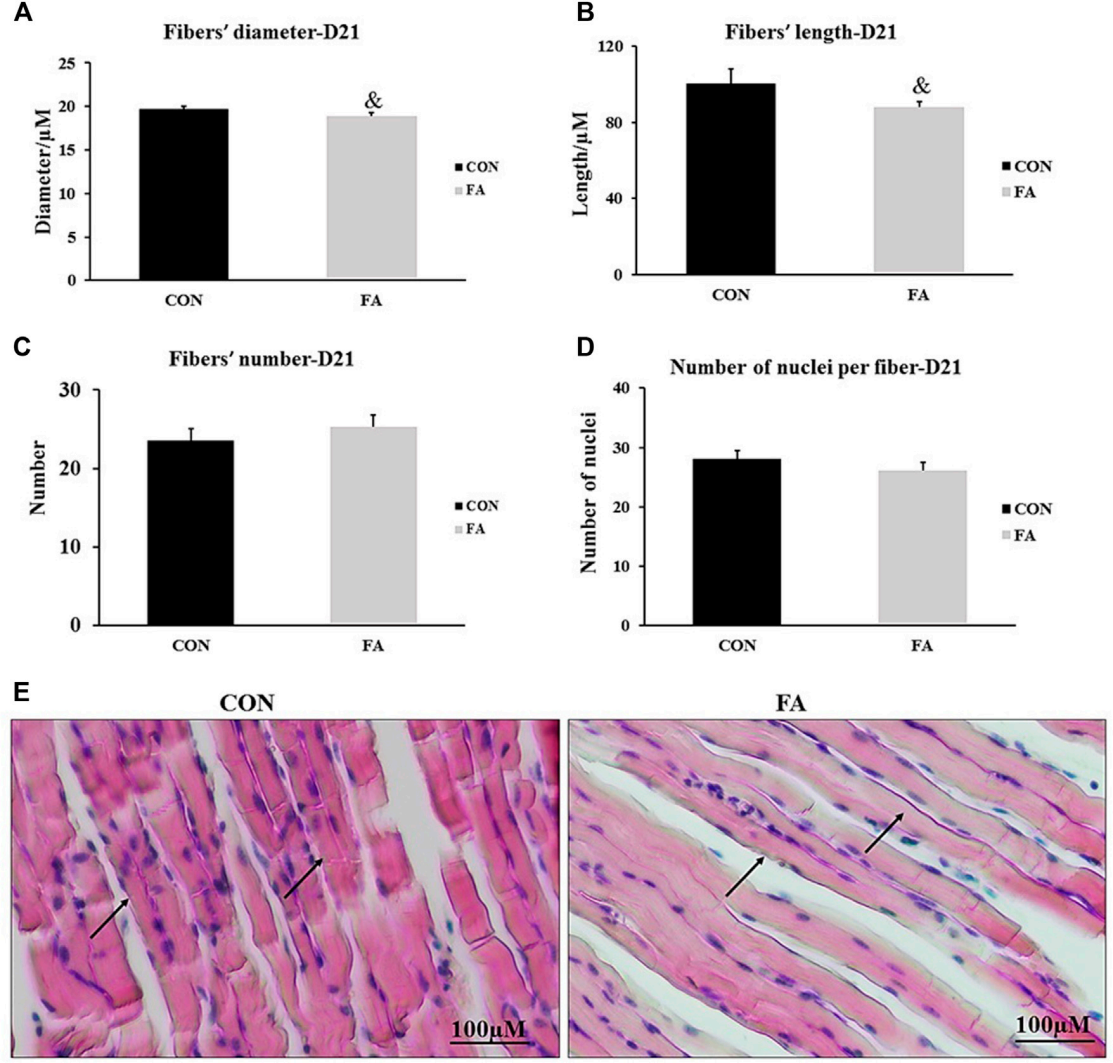
Effect of maternal intake of EPA/DHA on myotubes formation in day 21 weaned mice. The mothers were fed either control diet (CON) or EPA/DHA enriched diet (FA) during the entire period of pregnancy and lactation. **(A)** Data shows the difference in the diameter of muscle fibers measured in micrometer between CON and FA groups. **(B)** Data shows the difference in the length of muscle fibers measured in micrometer between control CON and FA groups. **(C)** The difference in the number of muscle fibers in the cross-sections between CON and FA treated groups. Images were analyzed in ImageJ-FIJI using the Freehand Selection Tool to encircle individual myofibers. **(D)** The difference in the number of nuclei per muscle fiber between CON and FA groups. The data represent as mean ± SEM. *p* < 0.05 (*n* = 8); eight samples per group were used in this measurement where the value of each sample represents the average of the measurements of 8 cross section areas randomly selected from each sample **(E)** a representative microscopic image of muscle tissue stained with H&E stain from differentially treated group. The magnification is ×10 and scale bar is 100 μm black arrows refer to muscle fibers. Non-significant difference was observed between groups.

The histological analysis of muscle tissue exhibited non-significant differences in the number of muscle fibers and number of nuclei per fiber between control and FA groups in weaned mice (*p* = 0.22 and 0.1). However, the results analysis of fibers’ diameter and length showed a tendency toward a decrease in FA group (18.89 ± 0.41 μM and 87.8 ± 3.02 μM respectively) when compared to control (19.96 ± 0.31 μM and 100.4 ± 7.8 μM respectively) (*p* = 0.074 and 0.096 respectively) (Figure 8).

### Intramuscular Fat Accumulation at D21

**FIGURE 9:**
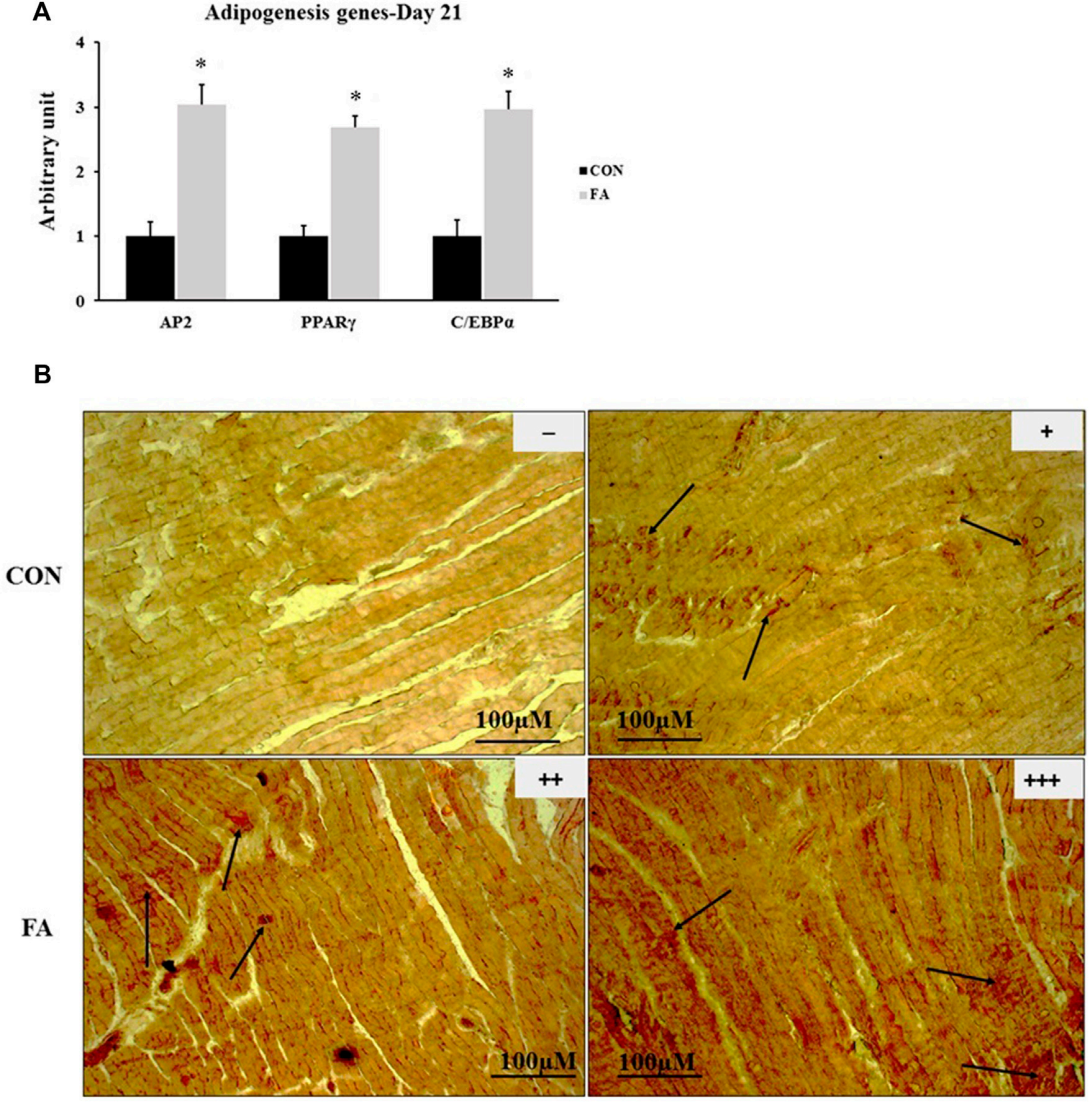
Effect of maternal EPA/DHA on ectopic lipid accumulation in muscles of day 21 weaned mice. The mothers were fed either control diet (CON) or EPA/DHA enriched diet (FA) during the entire period of pregnancy and lactation. **(A)** The relative expression of adipogenesis regulating genes. The raw Ct was normalized to the value of 18 s. All data represent as mean ± SEM. *p* < 0.05 by Student’s t-test (*n* = 6). **(B)** A representative microscopic image of muscle samples stained with Oil red O in day 21 offspring fed control diet or EPA/DHA enriched diet. The magnification is ×10 and scale bar is 100 μm. Black arrows refer to accumulated fat. (−) indicates absence the accumulation of fat neither in the sarcoplasm of the fibers nor in endomysium (between fibers). (+) represents mild fat infiltration. (++) indicates a moderate load. (+++) indicates an overload of fat.

The results exhibited a significant increase in the expression levels of PPARγ, CEBPα, and AP2, genes regulating the basal adipogenesis process in FA treated group when compared to control (169 ± 16.7%, *p* = 0.00001, 195 ± 29.1% *p* = 0.00023445, and 203 ± 30.9% *p* = 0.00013242 respectively). Correspondingly, a moderated to extensive increase in intramuscular and sarcoplasmic infiltration of lipid was observed in muscle samples stained with Oil Red O stain in group treated with EPA and DHA rich diet throughout the entire period of gestation and lactation when compared to control group (Figure 9).

### Genes Involved in Lipid Metabolism Regulation in Liver at D21

**FIGURE 10:**
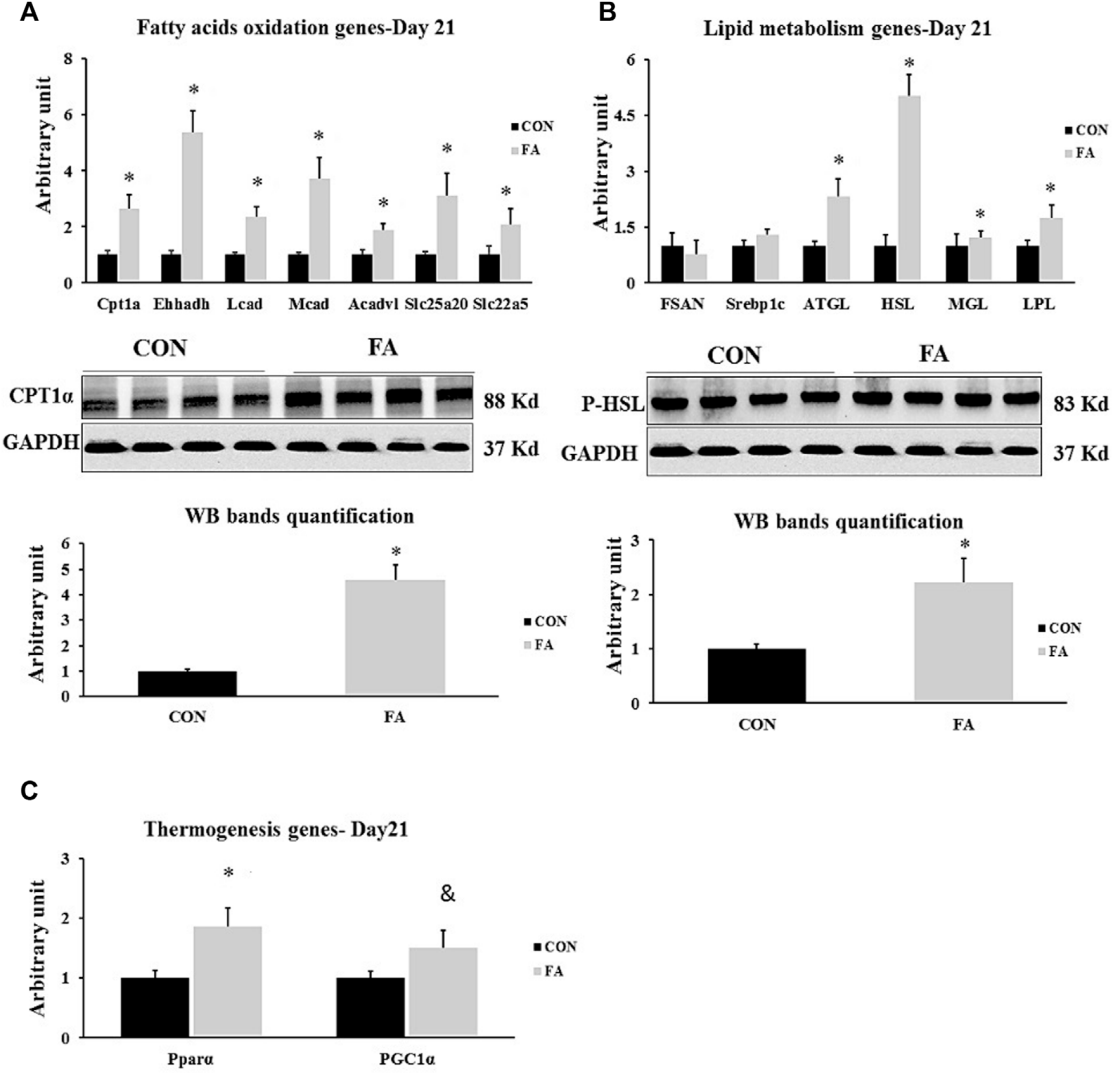
Effect of maternal intake of EPA/DHA on hepatic genes regulating lipid metabolism in weaned mice (D21). The mothers were fed either control diet (CON) or EPA/DHA enriched diet (FA) during the entire period of pregnancy and lactation **(A)** Quantitative RT-qPCR (*n* = 8) and representative image and densitometric analysis of western blot (*n* = 4) of genes regulating fatty acid uptake and a β-oxidation in neonates in weaned mice (D21) **(B)** Quantitative RT-qPCR (*n* = 8) and representative image and densitometric analysis of western blot (*n* = 4) of genes involved in regulating lipogenesis and lipolysis in weaned mice (D21). **(C)** Quantitative real-time PCR (*n* = 8) analysis of the expression of genes regulating the thermogenesis process in weaned mice (D21). The control image of GAPDH from liver sample (D21) was reused in panel B and **(C)**. The raw Ct was normalized to the value of 18 s. All data represent as mean ± SEM. *p* < 0.05 by Student’s t-test.

Our data revealed that after the treatment with EPA and EHA rich diet during gestation and lactation, there were significant changes of the expression levels of the genes involved in β-oxidation and thermogenesis in the offspring at D21. Considerable increase in the transcripts of CPT1α, Ehhadh, Mcad, Lcad, Acadvl, Slc22a5, Slc25a20, and PPARα was clearly observed in EPA and DHA treated group compared to control; whereas, the expression of PGC1α showed tendency toward an increase in maternal FA group (165 ± 49.8% *p* = 0.003204476, 436 ± 78.2% *p* = 0.00004, 272 ± 76% *p* = 0.001566, 133 ± 39.5% *p* = 0.002697168, 86 ± 24.3% *p* = 0.006101698, 109 ± 54.2% *p* = 0.046671763, 210 ± 81.3% *p* = 0.011363229, 86 ± 30.2% *p* = 0.008212401, and *p* = 0.069331773 respectively). Our findings exhibited that EPA and DHA have no effect on fatty acid synthesis in liver even though the treatment was sustained throughout the lactation period. In this regard, non-significant differences between tested groups in genes orchestrating lipid synthesis such as Fasn and Srebp1c (*p* = 0.32 and 0.114996 respectively) were detected between groups. However, lipolysis regulation genes including: ATGL and HSL, MGL and LPL (232 ± 46.3% *p* = 0.0072, 402 ± 57.6% *p* = 0.00001, 52 ± 21.4% *p* = 0.048, and 73.5 ± 34.4% *p* = 0.03 respectively) were significantly upregulated in FA treated group (Figure 10)**.**

### Brown Adipogenesis at D21

**FIGURE 11:**
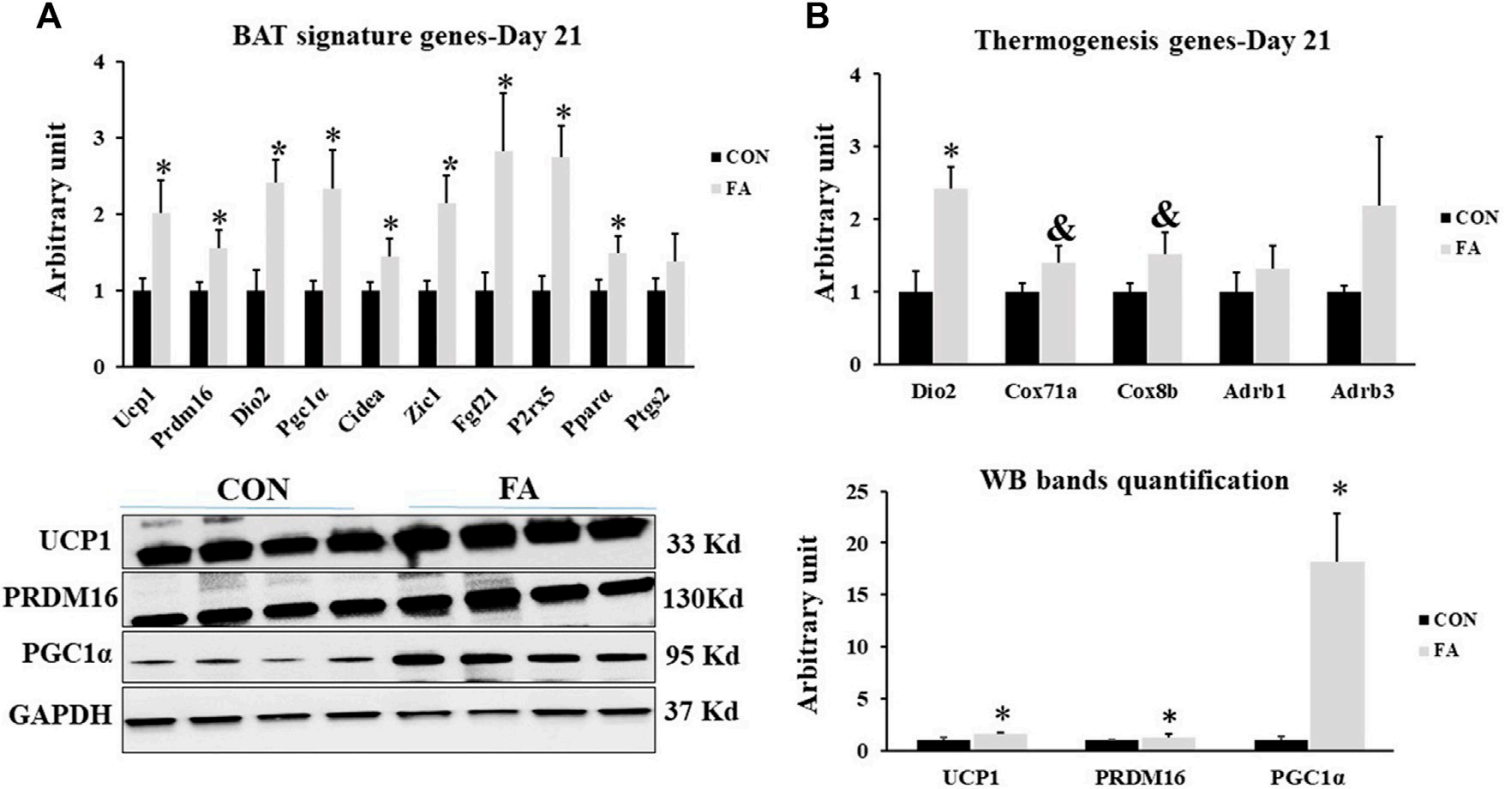
Effect of maternal intake of EPA/DHA on BAT signature genes expression in weaned mice (D21). The mothers were fed either control diet (CON) or EPA/DHA enriched diet (FA) during the entire period of pregnancy and lactation **(A)** Quantitative RT-qPCR (*n* = 8) and representative image and densitometric analysis of western blot (*n* = 4) of key genes regulating BAT activity and development in weaned mice, 3 weeks postpartum **(B)** The Quantitative RT-qPCR (*n* = 8) and representative image and densitometric analysis of western blot (*n* = 4) relative expression of genes involved in regulating the thermogenesis activity in weaned mice, 3 weeks postpartum. The raw Ct was normalized to the value of 18 s. All data represent as mean ± SEM. *p* < 0.05 by Student’s t-test.

The mRNA and proteins levels of BAT signature genes were measured to identify the effect of maternal n-3 PUFA supplementation on offspring’s BAT development and activity. Significant increase was observed in transcripts of uncoupling protein 1(Ucp1), cell death-inducing DNA fragmentation factor α-like effector A (Cidea), PR domain containing 16 (Prdm16), peroxisome proliferator-activated receptor gamma coactivator 1-alpha (PGC1α), type II iodothyronine deiodinase (Dio2), zinc finger protein ZIC 1 (Zic1), fibroblast growth factor 21 (Fgf21), P2X purinoceptor 5 (p2rx5), and peroxisome proliferator-activated receptor alpha (PPARα) in FA treated group compared to control (101 ± 42.5% *p* = 0.03, 45 ± 23.1% *p* = 0.04, 55 ± 23% *p* = 0.02, 133 ± 50.2% *p* = 0.01, 141 ± 30.4%, *p* = 0.002, 114 ± 35.6% *p* = 0.004, 182 ± 75.8% *p* = 0.019, 172 ± 42.2% *p* = 0.03, 49 ± 22.3% *p* = 0.03 respectively). Then, we assessed the effect of FA treatment on genes known to serve as stimulators of BAT thermogenic capacity. The results revealed an increase in the expression levels of Dio2, and PGC1α while a tendency toward an increase in the expression levels of cytochrome c oxidase polypeptide 7A1 (Cox7α1), and cytochrome c oxidase subunit 8B (Cox8β) was detected in FA treated group in comparison to control group (141 ± 30.4%, *p* = 0.002, 133 ± 50.2% *p* = 0.01, *p* = 0.076615, and 0.065654 respectively). However, no significant differences were observed in the expression levels of β3-adrenergic receptor (Adrb1) and β1-adrenergic receptor (Adrb3) between control and EPA/DHA treated groups (*p* = 0.117 and 0.22 respectively) (Figure 11).

### Potential Browning of Sub-Cutaneous White Adipose Tissue at D21

**FIGURE 12:**
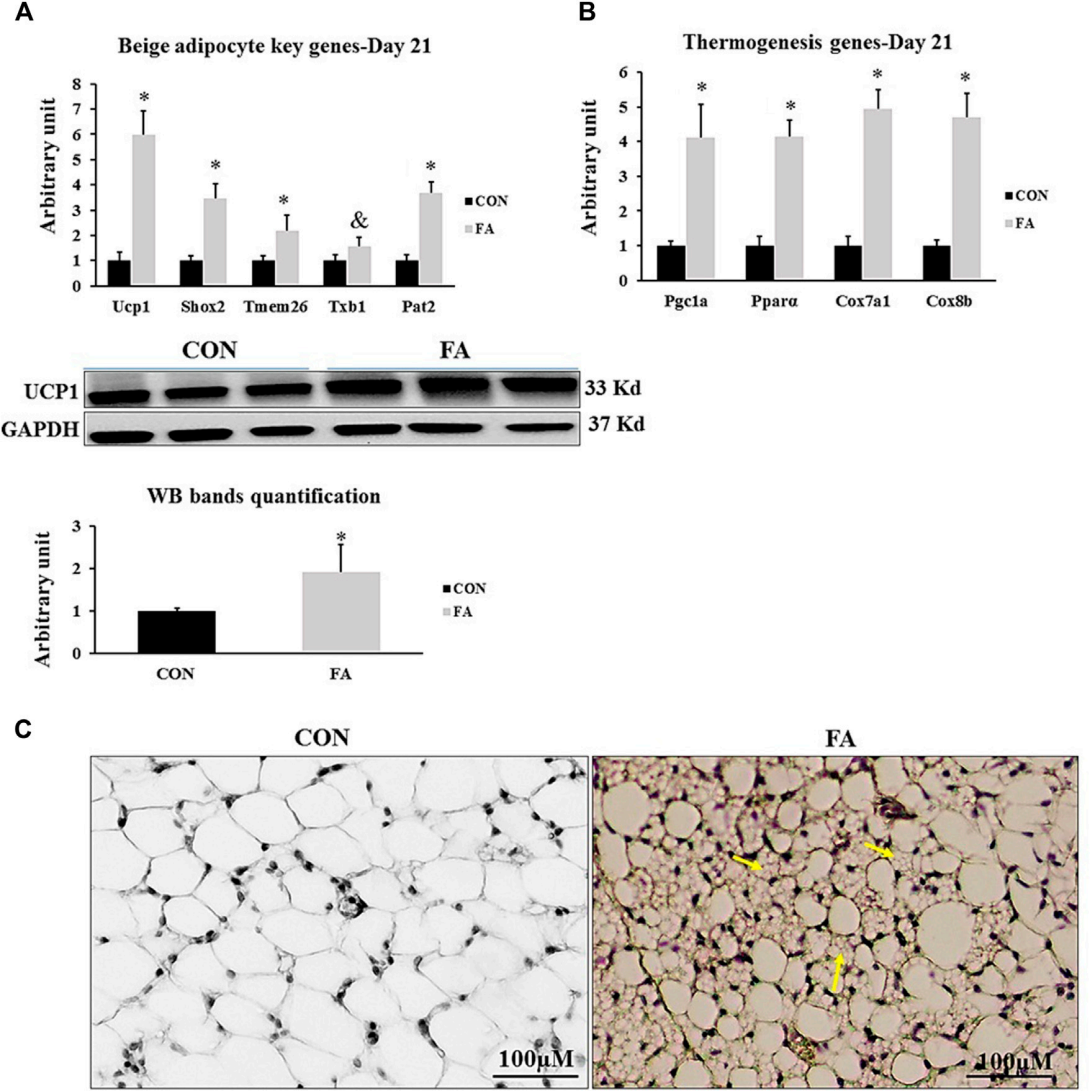
Effect of maternal intake of EPA/DHA on potential browning of subcutaneous fat in weaned mice (D21). The mothers were fed either control diet (CON) or EPA/DHA enriched diet (FA) during the entire period of pregnancy and lactation **(A)** Quantitative RT-qPCR (*n* = 8) and representative image and densitometric analysis of western blot (*n* = 4) genes regulating beige adipocytes development in sub-cutaneous fat of weaned mice, 3 weeks postpartum **(B)** The relative expression (*n* = 8) of genes involved in regulating the thermogenesis process in weaned mice, 3 weeks postpartum. All the raw Cts was normalized to the value of 18 s. Data represent as mean ± SEM. *p* < 0.05 by Student’s t-test. **(C)** A representative microscopic image of browning of sub-cutaneous fat stained with H&E. Yellow arrows refer to emerging beige adipocytes. The magnification is ×10 and scale bar is 100 μm.

The response of the mice to EPA and DHA supplementation was confirmed by assessing its effect on potential browning of sub-cutaneous fat. Beige fat gene profile was compared between differentially treated groups. Our analysis indicated a significant increase in the expression levels of beige specific markers such as Ucp1, Short-stature homeobox 2 (Shox2), transmembrane protein 26 (Tmem26), and Phosphate acetyltransferase (Pat2) and a tendency toward an increase in the expression levels of T-box transcription factor 1 (Txb1) (500 ± 92.2% *p* = 0.0001, 244 ± 60.7%, *p* = 0.0009, 116 ± 63.6% *p* = 0.050, 267 ± 43.2% *p* = 0.00004, and *p* = 0.09 respectively). Also, the expression levels of genes serving as stimulators of thermogenesis system, including PGC1α, PPARα, Cox7α1, and Cox8β were remarkably upregulated in response to EPA and DHA treatment (311 ± 95.6% *p* = 0.003, 414 ± 46.7% *p* = 0.00002, 393 ± 56.2% *p* = 0.00001, and 469 ± 68.3% *p* = 0.0001 respectively) (Figure 12).

### Fat Pad (Peri-Renal Fat) at D21

**FIGURE 13:**
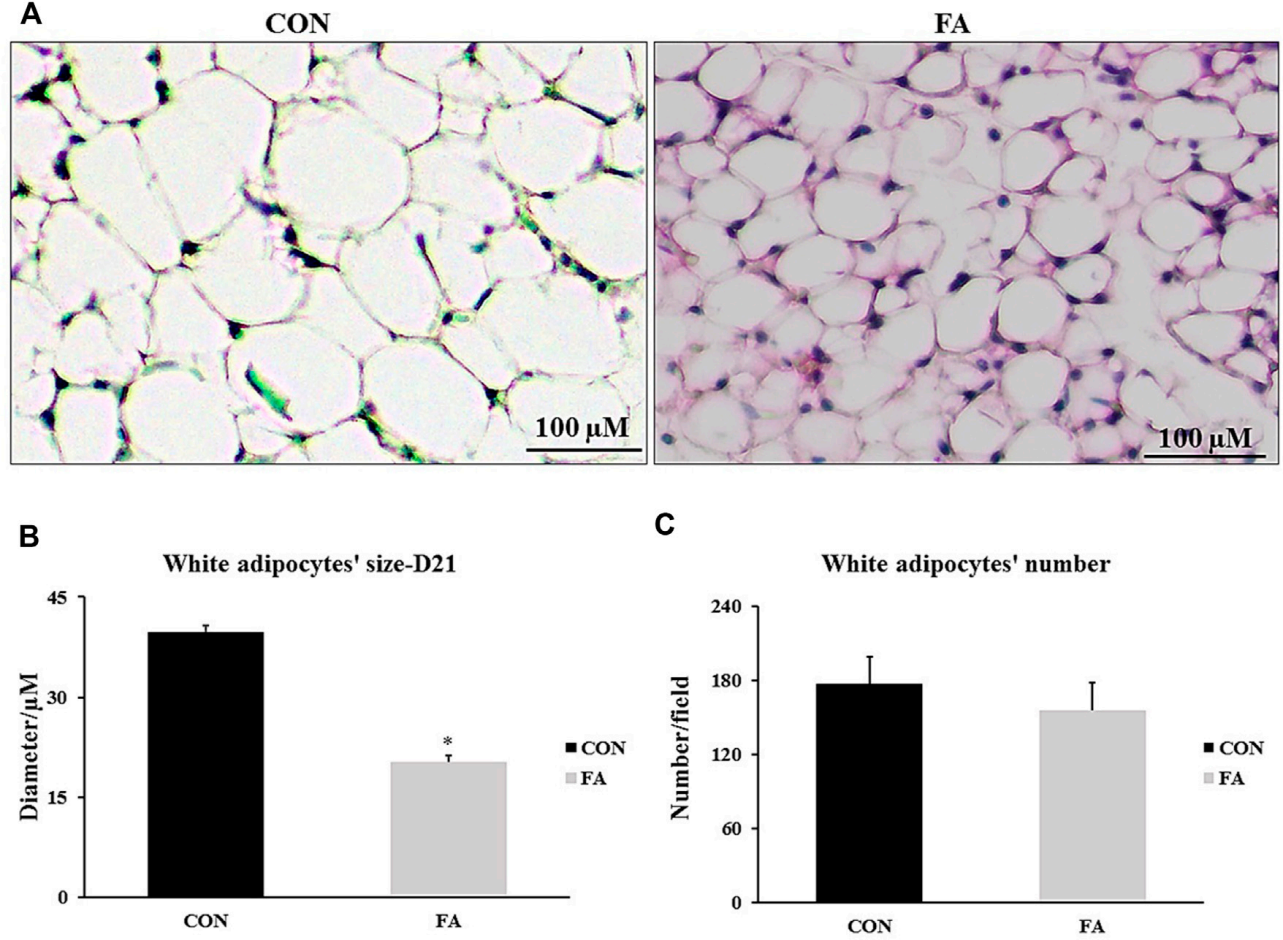
Effect of maternal intake of EPA/DHA on peri-renal fat pads of 21-day-old mice. The mothers were fed either control diet (CON) or EPA/DHA enriched diet (FA) during the entire period of pregnancy and lactation. **(A)** A representative microscopic image of H&E stain sections from each group. The magnification is ×10 and scale bar is 100 μm. **(B)** The Difference in adipocytes size measured in micrometer. **(C)** The differences in adipocyte number between different groups. Data represents as mean ± SEM. *p* < 0.05 by Student’s t-test (*n* = 8 pups).

The average adipocyte size was reduced in response to FA treatment (20.32 ± 0.9 μM) when compared to control (39.76 ± 0.92 μM) (*p* = 0.0001). However, adipocytes number was comparable between differently treated groups (*p* = 0.248) indicating that EPA/DHA treatment reduced adipocytes hypertrophy but has no effect on white adipocyte differentiation (hyperplasia) (Figure 13).

## Discussion

Anticipating its wide array of health benefits on offspring, maternal n-3 PUFAs intake has been frequently addressed as an area of investigation by researchers (Egert et al., 2009; Tateishi et al., 2009; Wang et al., 2013; Zhao et al., 2018). However, conducting a comprehensive study investigating the impact of n-3 PUFAs particularly EPA and DHA on fetal muscle development and energy handling is still missing. Here, we demonstrated that maternal supplementation of EPA and DHA induced excessive infiltration of intramuscular fat through up-regulation of PPARγ and other adipogenesis regulatory genes. However, the adipogenic effect was not on the expense of myoblasts as having been reported by several *in vitro* studies (Peng et al., 2012; Hsueh et al., 2018; Ghnaimawi et al., 2019; Zhang et al., 2019; Ghnaimawi et al., 2020; Lacham-Kaplan et al., 2020; Ghnaimawi et al., 2021). Otherwise, it was mediated by PPARγ activation, ectopically expressed in muscle cells. Moreover, no association was detected between EPA and DHA treatment and myotubes formation although it induced transient up-regulation of myogenesis regulating genes in neonates. The results also revealed that maternal supplementation of EPA and DHA could play a significant role in up-regulation the expression of genes regulating glucose metabolism indicating its inverse effect to insulin resistance. However, EPA and DHA maternal ingestion has no effect on gene expression and signaling related to protein synthesis even the treatment is sustained throughout the gestation and lactation period. Further, our findings documented the significance of maternal consumption of EPA and DHA enriched diet in promoting BAT development and thermogenic capacity, enhancing substrate oxidation in liver, stimulating white adipose tissue browning, and reducing adipocytes size.

The effect of maternal ingestion of EPA and DHA on myoblasts differentiation and myotube formation has been frequently addressed *in vitro* using C2C12 as a representative model of skeletal muscle cells with obvious inconsistency in the results. In relation to that, some investigations have asserted the beneficial effect of EPA and DHA in enhancing muscle strength and promoting the differentiation process (Dupont et al., 2019; Rodacki et al., 2012; Magee et al., 2008; Saini et al., 2017). Constantly, other studies have reported a negative association between EPA and DHA supplementation and pathways regulating myogenesis and terminal differentiation of myoblasts into mature myotubes (Ghnaimawi et al., 2019; Hsueh et al., 2018; Peng et al., 2012; Zhang et al., 2019; Lacham-Kaplan et al., 2020; Ghnaimawi et al., 2021; Ghnaimawi et al., 2020). Per our knowledge, this the first study investigating the effect of maternal diet enriched with EPA and DHA on fetal muscle development especially during the prenatal stage when *de novo* myogenesis process is initiated and during early postnatal stage when fully mature myofibers are formed. Our *in vivo* results were inconsistent with many *in vitro* studies including our previous ones which referred to the potential differentiation of C2C12 myoblasts into white adipocytes like phenotypes (Ghnaimawi et al., 2019; Hsueh et al., 2018; Peng et al., 2012; Zhang et al., 2019; Lacham-Kaplan et al., 2020; Ghnaimawi et al., 2021; Ghnaimawi et al., 2020). A transient increase in the expression levels of, MtoD1, MRF4, MyoG, and MHC4 was detected in the group fed EPA and DHA enriched diet at D1 (Figure 3) without any change in muscle mass (Figure 4). However, although it was not significantly different, EPA and DHA treated group exhibited a decrease in the expression levels of myogenesis regulatory transcription factors at E13 of gestation (Figure 2). The reason behind missing the correspondence between *in vivo* and *in vitro* trials can be attributed to one fact that is the proposed inhibitory effect of maternal EPA and DHA on myogenesis and myotube formation might be antagonized by the high concentration of reproductive hormones, mainly, 17β-estradiol (E2) during pregnancy. E2 is a strong stimulatory factor of myoblast differentiation and subsequent formation of full mature muscle fibers (Galluzzo et al., 2009; Murray and Huss, 2011; Berio et al., 2017; Lacham-Kaplan et al., 2020). It was reported that E2 plasma level during pregnancy is 100 times higher than EPA and DHA (Schock et al., 2016; Berkane et al., 2017) and n-3PUFA circulating level is 10 times less (Kawabata et al., 2017). Accordingly, we think that the inhibitory effect of EPA and DHA on myotube formation was overturned as a result of the considerable reduction in EPA and DHA to E2 ratio during pregnancy**.** Moreover**,** the transient postnatal increased in the expression of MRFs genes, MHC4, and IGF-1 in FA treated group observed in our study can be strongly linked to the stimulatory effect of E2 on the genes involved in myotubes formation and contractile proteins synthesis. Also, it may be a compensatory response to cope with the negative intervention of EPA and DHA against myogenesis during prenatal stage (E 13) and associated tendency toward decrease the expression levels of MyoD1 and MyoG, observed in this report. In accordance with that, the process of postnatal fully compensation of compromised muscle growth in response to maternal nutrient restriction or other inhibitory factors have been frequently addressed in human and animal trials (Jimenez-Chillaron and Patti, 2007; Tudehope et al., 2013).

EPA and DHA - mediated mTOR phosphorylation enhance the activation of P70-S6K1, followed by promoting protein synthesis and muscle fibers hypertrophy (Drummond et al., 2009; Baar and Esser, 1999). This pathway is the main regulatory route of muscle protein synthesis. Also, EPA and DHA are affective factors in up-regulating the expression of IGF-1, muscle protein synthesis stimulating factor (Wei et al., 2013). Although IGF-1 was significantly upregulated in FA treated group in comparison to control at D1 and 21, no change in muscle mass was observed. Also, EPA and DHA treatment has no effect on genes orchestrating muscle protein synthesis and degradation. Our results are consistent with other studies in which the basal rate of muscle protein synthesis was not affected by omega-3 supplementation (Smith et al., 2011a; Smith et al., 2011b; McGlory et al., 2016; Da Boit et al., 2017). EPA and DHA supplementation alone may not be sufficient to stimulate muscle protein synthesis which may reflect the divergence in the results. However, it can considerably potentiate the effectiveness of protein synthesis stimulated by dietary interventions such as in case of hyperinsulemia and hyper aminoacidemia. In line with such hypothesis, Tipton et al. (1999), Smith et al. (2011a), and Smith et al. (2011b) have reported that co-supplementation of omega-3 (1.86 g EPA, 1.5 g DHA/day) and amino acids for 8-week was effective in enhancing muscle mass expansion.

Our data indicated a promising role for EPA and DHA in reducing the potential incidence of insulin resistance in offspring by up-regulation the expression level of genes involved in orchestrating glucose metabolism such as insulin receptor (ISR) and IGF-1 but not Glut-4 in muscle samples culled at D 1 and 21 post-parturition (Figures 3, 5 respectively). According to Lanza et al., 2013, a significant increase in the expression levels of ISR and GLUT-4 were detected when omega-3 was added to high fat diet in mice. Other studies also have demonstrated increasing GLUT-4 expression only at the transcriptome level without providing further information about the protein level in response to omega-3 treatment (Figueras et al., 2011; Vaughan et al., 2012).

Our results also showed a dominant increase in the expression levels of adipogenesis regulating genes including PPARγ AP2, and CEBP/α at D1 and 21 with moderate to considerable infiltration of intramuscular fat in mice born from mothers fed fish oil enriched with EPA and DHA (Figures 6, 10 respectively). It is apparent that intramuscular fat accumulation observed in this animal model is mediated by stimulating the expression of the key regulators of adipogenesis process at the molecular level. EPA and DHA and eicosanoids are strong activators of PPARγ, the master gene involved in adipogenesis regulation (Tontonoz et al., 1998; Rosen et al., 2000; Dammone et al., 2018). It has been mentioned that up-regulating the expression of PPARγ is indispensable for the successful committing of Pre-adipocytes into mature adipocytes with full capacity of triglyceride synthesis and storage (Spiegelman, 1998; Feige et al., 2006; Tontonoz and Spiegelman, 2008)**.** Moreover, enhancement of fatty acid uptake to be utilized in triglyceride synthesis and lipid droplets formation *in vitro* requires increasing the expression of PPARγ and CEBP/α (Hu et al., 1995; Hu et al., 2012). Another proposed mechanism for increasing intramuscular lipid trapping in association to EPA treatment was linked to increasing the expression levels of GLUT1 and CD36/FAT (Aas et al., 2006). It has been recently demonstrated that ectopic lipid infiltration is a normal physiological process stimulated once muscle tissue exposed to damaging injury. The process is turned on after immediate muscle injury and last for few days before being stopped at the terminal stage of muscle repairing (Wagatsuma, 2007; Pisani et al., 2010; Pagano et al., 2015). Studies have provided evidence of the vital participation of PPARγ in regulating the regeneration process (Lukjanenko et al., 2013; Dammone et al., 2018). Additionally, it was asserted that glucose disposal could be improved once PUFAs was added to intravenously infused lipid. EPA induced incorporation of fatty acids into triglyceride synthesis was a beneficial in boosting muscle insulin sensitivity as it prevented the accumulation of deleterious lipid intermediates such as diacylglycerol and ceramides (Barber et al., 2013). Taken together, we can conclude that EPA and DHA could be classified as adipogenic factors capable of turning on a set of genes implicated in promoting the adipogenesis process and increasing intramuscular lipid accretion. Apparently, EPA and DHA exert their effect through activating the gene PPARγ that is ectopically expressed in muscle tissue. Considering the findings of aforementioned studies linking usefulness of omega-3 in improving insulin sensitivity and muscle regeneration, we think that EPA and DHA supplementation-induced intramuscular lipid overload, detected in this study, could not be marked as a deleterious process especially there was no change in integrity of muscular tissue. Instead, an increase in glucose metabolism regulating genes was documented. It is to be suggested that intramuscular accumulation of lipid is a physiological process regulated at the molecular level and might be switched on and/or off to confer a plasticity on muscle tissue to cope with surrounding environment.

Our results detected a considerable increase in the expression level of β-oxidation and thermogenesis regulation genes in liver at D21 only in FA group (Figures 10A,C). Also, up-regulation the expression levels of lipolysis regulating genes independent of changing the RNA transcripts of adipogenesis stimulating genes was observed in FA treated group (Figure 10B). The effect of EPA and DHA on lipid catabolism observed in our results showed a great similarity with other studies. It was reported a positive correlation between EPA and DHA consumption and activation of the transcription factor PPARα, followed by subsequent stimulation of lipolysis, fatty acid breakdown, and excessive production of energy in liver tissue (Clarke, 2001). However, this study is partially inconsistent with our data as it stressed an inhibition in lipogenesis regulating genes. Additionally, researchers found an increased the expression of PPARα, CPT-1α and CPT-2, the key regulators of β-oxidation, in response to fish oil dietary intervention (Yang et al., 2020). Further, EPA and DHA induced suppressing lipid synthesis and improving mitochondrial dysfunction in patient with liver steatosis has been frequently addressed *in vivo* and *in vitro* studies (Pachikian et al., 2011; Zhang et al., 2011). All the aforementioned experimental evidences have been demonstrated in adults while our findings demonstrated the same beneficial role of EPA and DHA in improving lipid catabolism in postnatal and weaned offspring. Taken together, EPA and DHA supplementation throughout the entire period of gestation is not sufficient to promote energy expenditure and preventing lipid accumulation in liver. Instead, sustained ingestion of EPA and DHA enriched diet during the period of pregnancy and lactation exhibited a great effectiveness in stimulating lipolysis, fatty acids uptake and oxidation, thermogenesis and mitochondrial function. In other words, maternal intake of omega-3 especially EPA and DHA could provide long-term metabolic benefits and improve lifespan once extended along the period of pregnancy and lactation. Moreover, maternal EPA and DHA promoted liver lipolysis independent of affecting the lipogenesis process.

Finally, we investigated the effect of EPA/DHA supplementation on fetal BAT development and activity and potential browning of subcutaneous white adipose tissue (sWAT). We found that maternal ingestion of EPA/DHA had no effect on BAT mass (Figure 1D). However, an increase in the expression and proteomic levels of master genes regulating brown adipogenesis was observed in FA treated group (Figure 11A) indicating the effectiveness of EPA and DHA in potentiating fetal BAT development and activity upon supplementation throughout pregnancy and lactation. Moreover, browning of sWAT (Figure 12) without changing adipose tissue mass was observed in response to maternal intake of EPA and DHA. The beneficial effect of EPA and DHA was mediated *via* increasing the expression of UCP1 and other genes involved in regulating the thermogenic capacity and mitochondrial biogenesis in subcutaneous fat, referring to the potential reprogramming of sub-cutaneous white adipocytes into beige adipocytes.

The observed effect of EPA and DHA on BAT development and activity in our study is in agreement with Fan et al. (2018) who reported that maintaining the ingestion of n-3 PUFA throughout the pregnancy and lactation was apparently associated with promoting BAT development and activity. They reported that the positive effect of EPA and DHA on BAT activity was conducted through activating their receptor, GPR120. In accordance with our results, the stimulatory effect of EPA and DHA on BAT specific genes in adult has been indicated in accumulative studies (Bargut et al., 2016b; Quesada-López et al., 2016; Pahlavani et al., 2017; Ghandour et al., 2018; Worsch et al., 2018; Bargut et al., 2019). The Thermogenesis process of BAT and sWAT can also be regulated by another two genes in addition to UCP1, including PGC1α and PPARα. PGC1α is the key gene responsible for orchestrating mitochondrial biogenesis by increasing the expression level of mitochondrial transcription factor A (TFAM) (Nadal-Casellas et al., 2013; Yu et al., 2015; Bargut et al., 2017). The effect of EPA and DHA on PGC1α expression in BAT and sWAT observed in our results exhibited a great similarity with previous reported researches (Pahlavani et al., 2017; Worsch et al., 2018; Bargut et al., 2019).

PPARα is another ligand of EPA and DHA through which they modulate body metabolism. PPARα activation has been linked to increasing mitochondrial content, fatty acids oxidation, and up-regulating UCP1 expression (Hondares et al., 2011; Barberá et al., 2001). EPA and DHA mediated up-regulation of PPARα in sWAT and BAT, reported in this study is corresponding to other studies (Bargut et al., 2016a; Bargut et al., 2016b; Bargut et al., 2019; Huang et al., 2016). However, EPA and DHA treated mice exhibited non- significant changes in the expression of β-adrenergic receptors (Figure 11B) despite of their importance in regulating the thermogenesis process. The results refer to the effectiveness of EPA and DHA intake in improving BAT thermogenic capacity independent of stimulating β-adrenergic receptors. Our results are inconsistent with other studies having reported an important role of EPA and DHA in stimulating the thermogenesis process through up-regulation of genes encoding β-adrenergic receptors synthesis (Kim et al., 2015; Fan et al., 2018; Ghandour et al., 2018). We may conclude that EPA and DHA mediated stimulation of β-adrenergic receptors could be dose and time dependent. Thus, prolonged exposure to EPA and DHA is requisite to induce Adb1 and Adb3 activation while maternal intake of EPA and DHA throughout gestation and lactation may not be sufficient to successfully carry out such stimulation.

Browning of sWAT can be verified by increasing the expression of UCP1 and other thermogenesis regulating genes and beige specific markers. Hereby, we found a considerable increase in the transcripts of beige specific markers (Figure 12A). Further, thermogenesis regulating genes were upregulated as well (Figure 12B). In line with our outcomes, the beneficial role of EPA and DHA in promoting the origination of beige adipocytes from sWAT has been addressed by several studies, indicating their participation at least to some degree in countering high fat induced obesity (Kim et al., 2015; Bargut et al., 2016a; Bargut et al., 2016b; Kim et al., 2016; Pahlavani et al., 2017). It was demonstrated that the effect of EPA and DHA could be mediated *via* stimulation of AMP-activated kinase (AMPK) (Lorente-Cebrián et al., 2009; Abdul-Rahman et al., 2016). According to previous studies, EPA and DHA exhibited an independency in their effect on brown and beige adipocytes where the beneficial effect was attributed to the presence of EPA (Zhao and Chen, 2014; Kim et al., 2016; Quesada-López et al., 2016). In accordance with that, the stimulatory effect of EPA and DHA enriched diet on BAT activity and browning of sWAT detected in our study might be related to the presence of EPA.

## Conclusion

We conclude that feeding fish oil enriched with EPA and DHA throughout gestation and lactation is associated with activating PPARγ and downstream basal adipogenesis regulating genes, AP2 and C/EBPα, followed by intramuscular accumulation of fat. A transient increase in the expression of genes regulating myogenesis and myosin heavy chain 4 detected in newborn mice and the dominant up-regulation of protein synthesis stimulating gene, IGF-1, throughout gestation and lactation were occurred without of inducing myotubes hypertrophy or hyperplasia. It is to be noticed that EPA/DHA supplementation-induced accumulation of fat between muscle fibers was not the consequence of the potential trans-differentiation of myoblast into adipocytes as it has been demonstrated by many *in vitro* trials. Instead, it was related to the ectopic activation of PPARγ, the key regulator of adipogenesis. Persistent increase in the expression of gene encoding ISR production was observed in offspring at the age of day 1 and 21 could be an indicator of improving muscle glucose metabolism. It is to be suggested that the exposure to maternal diet containing high ratio of EPA and DHA in comparison to n-6 fatty acids during pregnancy is sufficient only to induced partial increase in substrate oxidation regulating genes in livers of day 1 neonates. However, EPA and DHA treatment throughout gestation and lactation showed a great effectiveness in promoting the expression of fatty acids uptake, β-oxidation, thermogenesis, and lipolysis orchestrating genes independent of changing the lipogenesis process. Boosting brown adipogenesis transcriptional programme and promoting the potential browning of sWAT were closely linked to sustained ingestion of EPA/DHA during pregnancy and lactation. An increasing the thermogenesis capacity of EPA/DHA treated group was accompanied with decreasing the weight of weaned mice although it was not significant. Maternal intake of fish oil has no effect on body weights and number of births in day 1 newborns. Based on our observations, EPA and DHA ingestion during pregnancy and lactation can be suggested as a therapeutic strategy to improve lipid metabolism and combat childhood obesity induced metabolic disorders.

## Data Availability

The original contributions presented in the study are included in the article/supplementary materials, further inquiries can be directed to the corresponding author.
